# Sulfate Attack-Induced C-S-H Gel Degradation Mechanism and Machine Learning-Based Strength Prediction of Coal Gangue Aggregate Concrete

**DOI:** 10.3390/gels12080712

**Published:** 2026-08-11

**Authors:** Shuanghua He, Ruicong Han, Junfeng Guan, Ying Hao, Li Zhao, Yafei Jing

**Affiliations:** 1 School of Civil Engineering and Transportation, North China University of Water Resources and Electric Power, Zhengzhou 450045, China; 2 School of Water Conservancy, North China University of Water Resources and Electric Power, Zhengzhou 450046, China

**Keywords:** coal gangue aggregate concrete, sulfate attack, C-S-H gel degradation, mechanical properties, corrosion resistance coefficient, machine learning, prediction model

## Abstract

Coal gangue concrete (CGC) is an effective green building material that can promote the resource utilization of solid waste. To study its durability performance and degradation mechanism under sulfate attack with dry-wet cycles, and to realize the intelligent prediction of mechanical properties, this study prepared CGC specimens with a water-to-binder ratio of 0.4, a fine aggregate replacement rate of 20%, and coarse aggregate replacement rates of 0%, 20%, 50%, 80%, and 100%. The specimens were tested under 30, 60, 90, and 120 dry-wet cycles in 10% MgSO_4_ solution. Mass loss, relative dynamic elastic modulus, and compressive and flexural strength corrosion resistance coefficients were used as evaluation indices, and SEM and XRD were adopted to analyze microstructural deterioration. A database compiled from literature data was established, and six machine learning models-random forest (RF), artificial neural network (ANN), decision tree (DT), support vector machine (SVM), particle swarm optimization-artificial neural network (PSO-ANN), and particle swarm optimization-support vector machine (PSO-SVM) were constructed to predict the strength corrosion resistance coefficients. Test results indicate that all macroscopic indices first increased and then decreased with the number of dry-wet cycles. Early ettringite and gypsum products filled internal pores, while prolonged sulfate attack caused decalcification and structural degradation of C-S-H gel, resulting in obvious performance loss. The PSO-SVM model showed the best prediction accuracy, with R^2^ values of 0.912 and 0.981 for compressive and flexural strength corrosion resistance coefficients, respectively. Feature importance analysis shows that dry-wet cycles had the most significant negative impact, followed by the coal gangue fine aggregate replacement rate. This study provides support for the durability evaluation and intelligent prediction of coal gangue concrete in sulfate environments.

## 1. Introduction

Coal gangue is the primary solid waste generated during coal mining. Simple stockpiling or landfill disposal not only occupies substantial land resources but also causes severe environmental pollution. Given the similarity between coal gangue and natural aggregates, producing concrete with coal gangue is regarded as an effective approach for its large-scale utilization. Owing to the differences in physical properties between coal gangue and natural aggregates, replacing natural aggregates with coal gangue can influence concrete strength to varying degrees. Consequently, extensive research has been conducted by many scholars on the mechanical properties, workability, and microstructure of coal gangue concrete (CGC).

Zhang et al. [1] studied the mechanical and environmental properties of coal gangue sand concrete at different concrete strength grades (C30, C50) and aggregate replacement rates (0, 25%, 50%, 75%, 100%). The results showed that coal gangue sand weakened the bonding performance of the interfacial transition zone between cement mortar and coarse aggregate. Regardless of the concrete grade, the elastic modulus and axial compressive strength of coal gangue sand concrete exhibited a downward trend with the increase in replacement rate. Gao et al. [2] used coal gangue as a substitute for coarse aggregate in concrete and investigated its effects on the mechanical properties of concrete material, reinforced concrete cylinders, and concrete-filled steel tube cylinders. Their study indicated that the mechanical properties of CGC showed a decreasing trend as the coal gangue replacement rate increased. Zhou et al. [3] prepared concrete by replacing natural gravel (N) with spontaneous combustion coal gangue (S) or rock coal gangue (R), respectively, and conducted mechanical property tests. They found that although the failure modes of different types of CGC varied, the slope of the ascending branch of the stress–strain curve and the peak stress both decreased, while the peak strain increased. Yao et al. [4] carried out experimental studies on the static and dynamic properties of CGC and found that C30 and C40 CGC could be produced using coal gangue as both coarse and fine aggregates. Qiu et al. [5] discovered that an appropriate fly ash content could improve the mechanical properties of CGC. This was attributed to the fact that fly ash filled the internal pores of concrete during the early hydration of cement, and its secondary hydration could modify the microstructure of the mortar and the interfacial transition zone. Li et al. [6] found that compared with ordinary concrete, CGC had poorer workability and lower compressive strength. Wang Liang et al. [7] explored the influence of different coal gangue fine aggregate replacement rates (0%, 10%, 20%, 40%, 50%) on concrete strength and found that the concrete properties changed little when the replacement rate of coal gangue fine aggregate was below 20%. Jia et al. [8] investigated the effects of the water-to-binder ratio and fly ash on the compressive strength of CGC, finding that the maximum compressive strength was obtained at a water-to-binder ratio of 0.4 and a fly ash content of 10%. Sun Gangzhu et al. [9] studied concrete with different coal gangue coarse aggregate replacement rates (0%, 30%) and coal gangue fine aggregate replacement rates (0%, 10%, 20%, 30%, 40%, 50%), finding that concrete performance was optimal when the coal gangue fine aggregate replacement rate was 20%.

Although an appropriate CGC replacement ratio can improve the mechanical properties of concrete in the short term, the porous structure, compositional variability, and potentially reactive substances of coal gangue aggregates may pose challenges to the durability of concrete. Among numerous durability issues, sulfate attack is one of the most prevalent and detrimental factors leading to the deterioration of concrete structural performance and the shortening of service life. Sulfate ions undergo a series of physical and chemical reactions with cement hydration products, generating expansive products such as ettringite and gypsum, which induce substantial internal stress within the concrete, resulting in cracking, spalling, and loss of strength. In parallel, the calcium–silicate–hydrate (C-S-H) gel itself can undergo decalcification and structural degradation, directly impairing the matrix integrity. Although considerable research on the sulfate attack of CGC has been carried out by many scholars, the underlying C-S-H gel degradation mechanism remains insufficiently clarified, and reliable strength prediction approaches are still lacking.

Yang et al. [10] immersed mineral compositional, microstructural, functional group and elemental analyses were performed using X-ray diffraction technique, IR infrared diffraction, electron microscope observation, and energy spectrum analysis, to elucidate the mechanisms of the strength development. Yang et al. [11] employed a combination of X-ray diffraction (XRD), nanoindentation, and scanning electron microscopy–energy dispersive spectroscopy (SEM-EDS) techniques to investigate the evolution of the composition and mechanical properties of calcium aluminosilicate hydrate (C-A-S-H) gel in cement–slag paste under sulfate attack. Guo et al. [12] conducted sulfate resistance tests on CGC and found that it possessed a certain degree of sulfate resistance. Li Yongjing et al. [13] adopted an orthogonal test method to investigate the sulfate resistance of CGC under two conditions: long-term immersion and dry-wet cycles. The results indicated that the performance of CGC improved in the early stages of attack, while all properties decreased after prolonged erosion. Guan et al. [14] conducted rapid freeze-thaw tests on CGC with different volume replacement rates (0%, 20%, 40%, 60%) and tested the compressive strength. The results showed that the compressive strength and frost resistance of concrete decreased with increasing freeze-thaw cycles, and the deterioration rate was proportional to the coal gangue content.

The deterioration of CGC is governed by multiple factors with complex nonlinear interrelationships. Machine learning, owing to its strong regression and fitting capabilities, can effectively handle the nonlinear relationships among complex data and is highly compatible with the intricate material composition of CGC, and it has been widely applied in the field of civil engineering.

Zhao et al. [15] developed an XGBoost-based algorithm to predict the compressive strength of self-compacting concrete. The results indicated that using machine learning models to predict the strength of self-compacting concrete containing recycled aggregate had high accuracy. Furthermore, Chen et al. [16] used the random forest (RF) algorithm, least squares support vector machine (LSSVM), and the non-dominated sorting genetic algorithm II (NSGA-II) method to study the durability of high-performance concrete. Zhou et al. [17] established a neural network model to predict the compressive strength of fly ash concrete and found that neural network-based prediction of fly ash concrete compressive strength is feasible and achieves high prediction accuracy. Zhang et al. [18] established a neural network model to predict the compressive strength of concrete under sulfate attack and found that the model had small prediction errors and high prediction accuracy. Kuang Xiangwen et al. [19] established a neural network model to predict the compressive strength of concrete and found that the prediction error was less than 3%, yielding accurate prediction results. Zheng et al. [20] proposed two efficient machine learning models to evaluate the macroscopic mechanical properties of recycled coarse aggregate self-compacting concrete (RCASCC) after sulfate freeze-thaw action. Using stress–strain data from uniaxial compression tests, a machine learning model for RCASCC after sulfate freeze–thaw cycles was developed in MATLAB. Eight different machine learning algorithms were used to train and test the model, and six performance metrics were employed to measure its generalization performance. Three models—RF, ET, and GB—demonstrated the highest prediction accuracy compared with the other machine learning models.

However, current studies still have the following key limitations.

Regarding complexity of deterioration mechanisms versus singularity of research scenarios, most studies rely on long-term full-immersion tests. In real service environments, however, concrete is exposed to repeated dry-wet cycles coupled with sulfate attack. Such conditions are common in coastal areas and saline soils. Dry-wet alternation accelerates sulfate capillary absorption and salt crystallization damage. The combined effect of physical and chemical attack leads to more severe deterioration. Systematic research on this more realistic deterioration mechanism is still lacking.

With regard to qualitative and fragmented description of deterioration laws, many studies only compare macroscopic strength before and after erosion. They provide qualitative observations of microstructure. A quantitative description of the dynamic evolution among replacement ratio, erosion age, and mechanical properties remains absent.

There are concerns about the insufficient applicability and intelligence of prediction models. Traditional empirical or semi-empirical models cannot effectively handle the strong nonlinear coupling among coal gangue content, erosion cycles, and water-to-binder ratio. Machine learning has been applied to ordinary concrete, but no dedicated high-precision prediction model exists for CGC under sulfate dry-wet cycle erosion. The few related studies rely on a limited set of algorithms. They have not fully used ensemble learning or intelligent optimization algorithms to improve prediction accuracy and generalization, especially for small-sample, high-dimensional, and nonlinear problems.

This study focuses on the main line of “deterioration mechanism and performance prediction.” The research work is as follows.

Real service environments were simulated for quantitative study of time-varying mechanical properties. Concrete specimens with different coal gangue replacement ratios were prepared. Sulfate dry-wet cycle tests were conducted to better simulate real erosion conditions. Compressive and flexural strengths were tested systematically. A quantitative relationship linking replacement ratio, number of cycles, and corrosion resistance coefficient is established. This reveals the dynamic attenuation of mechanical properties under multi-factor coupling.

For multi-scale microscopic characterization and intrinsic correlation with macroscopic degradation, SEM and XRD were used to dynamically track the evolution of micro-morphology and phase composition during erosion. The analysis particularly focuses on deterioration of the interfacial transition zone (ITZ) under dry-wet cycling. The cross-scale damage mechanism is clarified; microcrack initiation and propagation caused by expansive products and salt crystallization pressure lead to macroscopic strength loss. This provides a physical basis for the prediction model.

High-precision strength prediction is based on multi-model comparison and intelligent optimization. Experimental and literature data were used to construct and compare six machine learning models: RF, ANN, DT, SVM, PSO-ANN, and PSO-SVM. Particle swarm optimization (PSO) was introduced to optimize the hyperparameters of traditional ANN and SVM models. This overcomes their tendency to fall into local optima. The best model was selected through multi-dimensional evaluation metrics. Feature importance analysis was used to enhance physical interpretability. An intelligent strength prediction method for CGC under sulfate dry-wet cycle erosion is established.

## 2. Results and Discussion

### 2.1. Analysis of Sulfate Attack Test Results

The apparent morphological changes of CGC specimens under the combined action of sulfate attack and dry-wet cycle are shown in Table 1.

#### 2.1.1. Analysis of Test Results

##### Mass Change of Specimens Under Sulfate Attack

The mass of the CGC specimens subjected to sulfate attack and different numbers of dry-wet cycles was measured, and the mass loss of the specimens was evaluated using the mass loss rate. The measured mass and calculated mass loss rates of the specimens are presented in Table 2 and Table 3.

**Figure 1:**
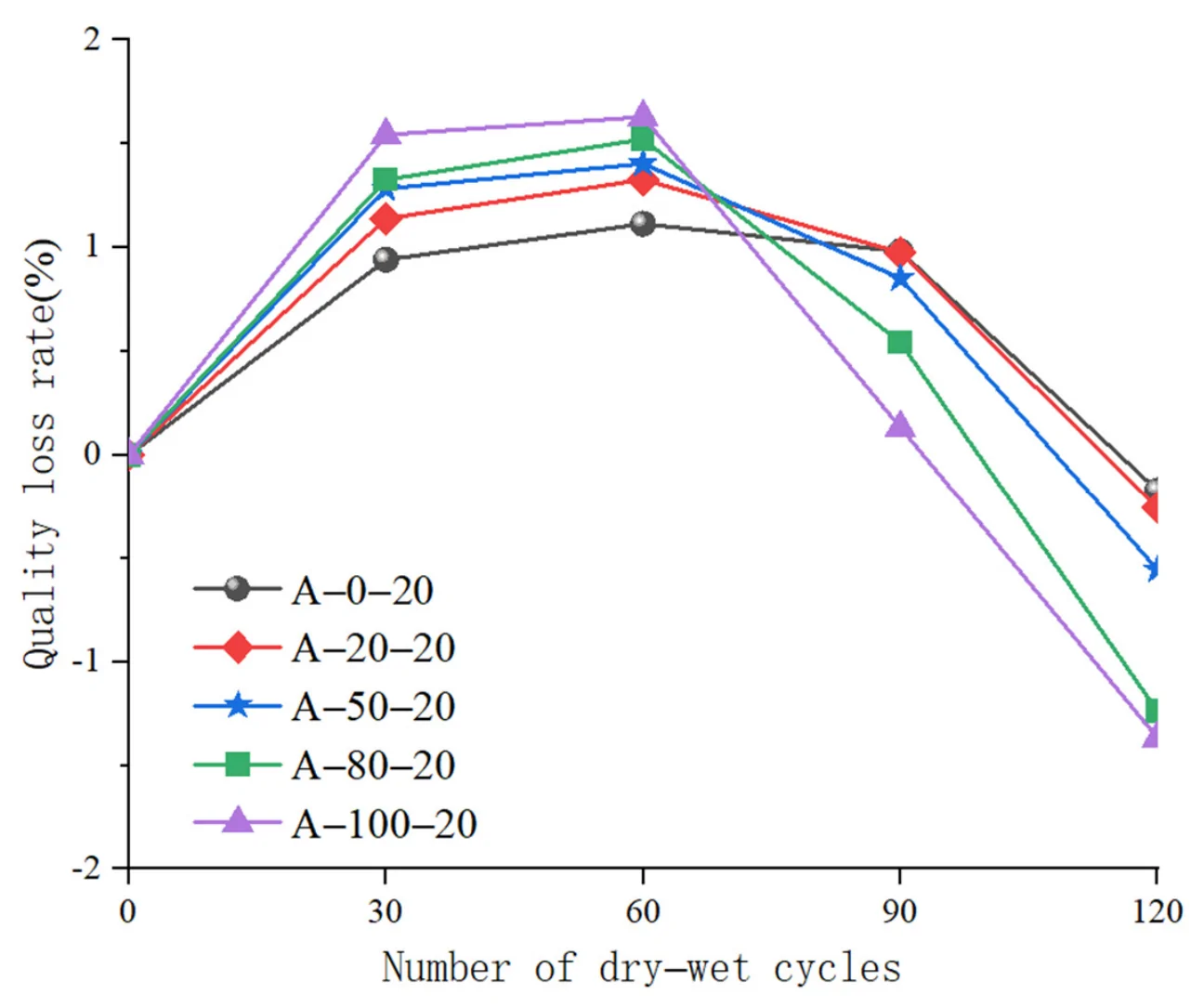
Variation of mass loss under different dry-wet cycles.

Figure 1 illustrates the variation pattern of the mass loss of CGC specimens under different numbers of dry-wet cycles. As shown in the figure, with the increase in the dry-wet cycling, the mass loss rate of the specimens first decreased and then increased. During the early stage of attack (0–60 dry-wet cycles), the mass of the specimens was in an increasing phase and reached the maximum at 60 cycles. This is because the SO_4_^2−^ ions diffusing from the solution reacted with cement hydration products to form ettringite (AFt) and other sulfate products, which filled the internal pores of the specimens and led to an increase in mass. However, as the dry-wet cycles progressed and the sulfate attack continued, expansive products accumulated persistently inside the specimens. At the same time, the decalcification and structural degradation of the C-S-H gel gradually undermined the integrity of the binder matrix. The increasing amounts of expansive products generated new cracks within the specimens, which caused the edges and corners to become loose and friable and to spall off. Consequently, the mass of the concrete specimens exhibited a downward trend in the later stage of attack, accompanied by severe mass loss.

##### Change in Relative Dynamic Elastic Modulus of Specimens Under Sulfate Attack

**Figure 2:**
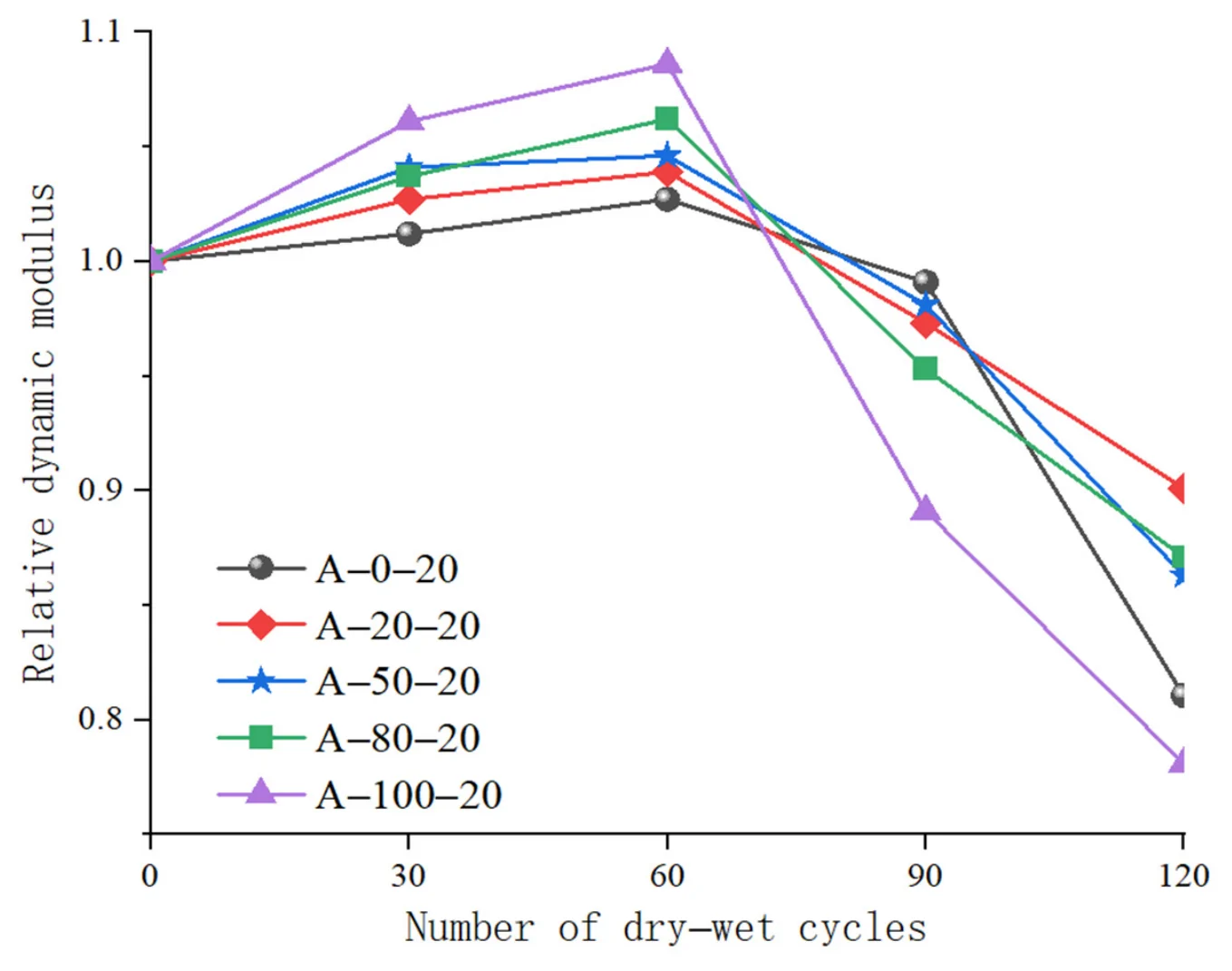
Relative dynamic modulus of CGC specimens under dry-wet cycles.

The relative dynamic elastic modulus of the specimens was calculated according to Equation (2), and the calculated results are presented in Table 4. The variation pattern of the relative dynamic elastic modulus of CGC under different numbers of dry-wet cycles is shown in Figure 2. Similar to the variation pattern of the mass loss rate, the relative dynamic elastic modulus of CGC under different dry-wet cycles can be divided into two stages: an increase stage and a decrease stage. The relative dynamic elastic modulus reached its peak at 60 dry-wet cycles and then gradually declined. In the early stages of sulfate attack, cement hydration products reacted with SO_4_^2−^ ions to generate expansive substances (ettringite) and sulfate crystals, which filled the internal pores of the concrete and densified the internal microstructure. Consequently, the relative dynamic elastic modulus increased gradually. With the continued action of sulfate attack and dry-wet cycles, the increasing amount of ettringite caused internal cracking of the specimens. As microcracks developed rapidly, the internal structure of the specimens transformed from dense to loose, and spalling began at the edges and corners. The relative dynamic elastic modulus decreased, and at this stage it was in a sharp decline phase.

##### Change in Compressive Strength Corrosion Resistance Coefficient Under Sulfate Attack

**Figure 3:**
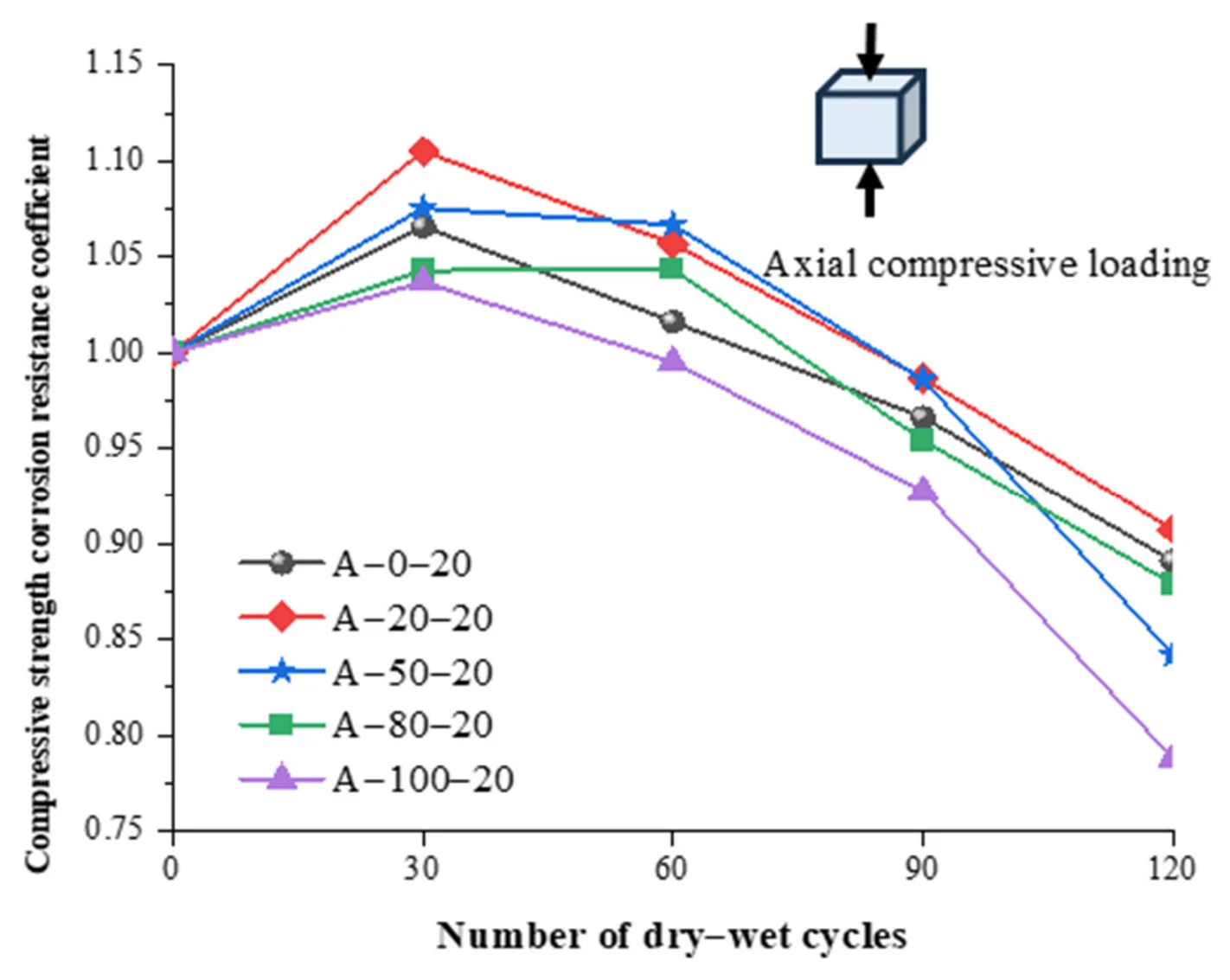
Compressive strength corrosion resistance coefficient under different numbers of dry-wet cycles.

The compressive strength results of the specimens subjected to different numbers of dry-wet cycles are presented in Table 5. The compressive strength corrosion resistance coefficient of CGC was calculated according to Equation (4), and the calculated results are presented in Table 6. Under the combined action of sulfate attack and different dry-wet cycle ages, the compressive strength corrosion resistance coefficient exhibited a trend of first increasing and then decreasing, as shown in Figure 3. In the early stages of dry-wet cycling, the products from cement hydration and sulfate attack jointly filled the internal pores of the specimens, which gradually densified the internal structure and further enhanced the sulfate resistance of the concrete. The compressive strength corrosion resistance coefficient reached its maximum value at 30 dry-wet cycles. However, as the number of dry-wet cycles increased and the sulfate attack continued, the persistent expansion of ettringite inside the specimens generated expansion stress. When the expansion stress exceeded the tensile strength of the concrete, new microcracks formed in the interior, altering the internal microstructure and causing cracking and spalling of the concrete, thus leading to a decrease in compressive strength. The coefficient gradually declined from 30 dry-wet cycles onward, and after 60 dry-wet cycles, it dropped sharply until the end of the erosion process.

##### Change in Flexural Strength Corrosion Resistance Coefficient Under the Coupled Action of Sulfate Attack and Dry-Wet Cycles

**Figure 4:**
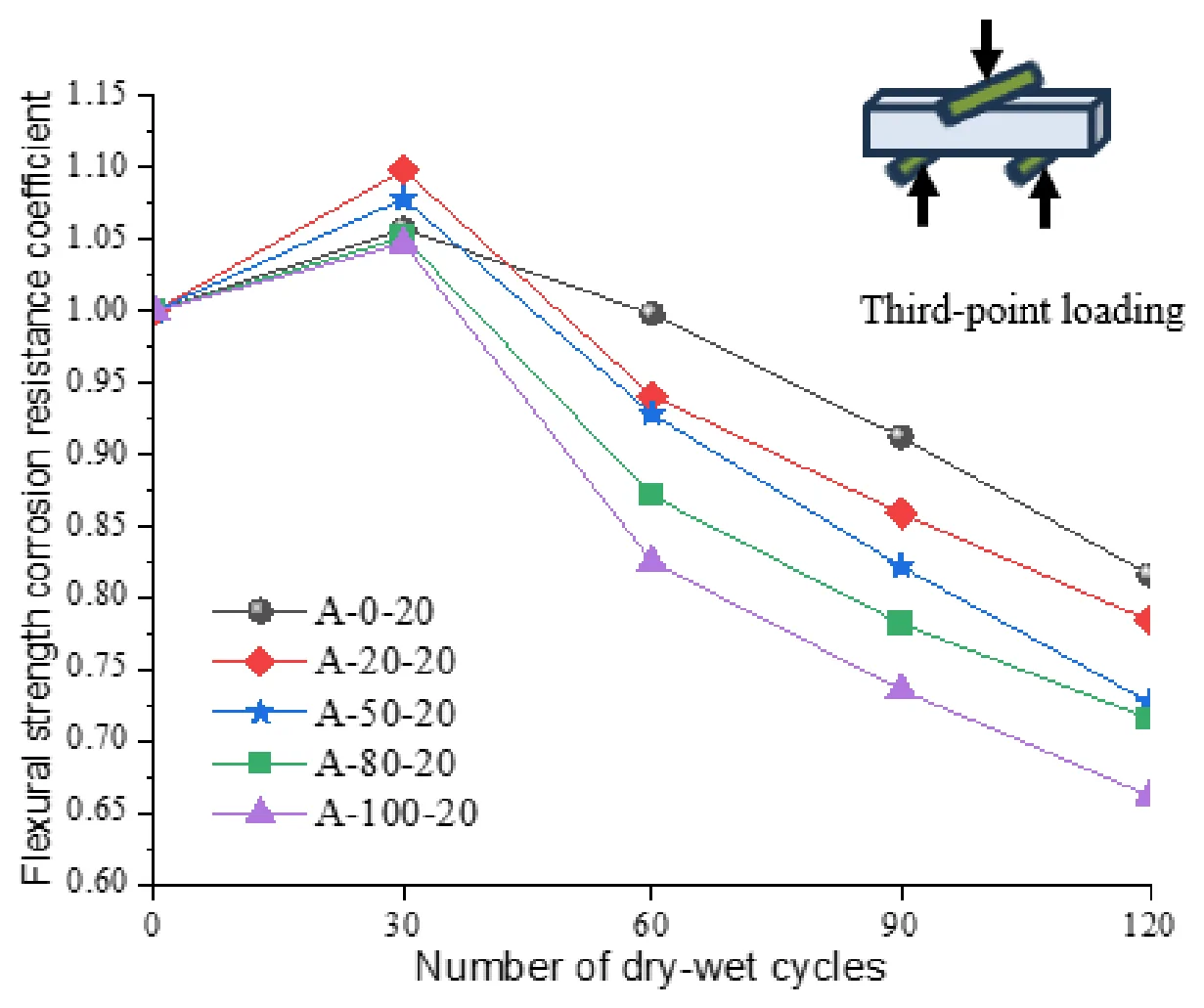
Flexural strength corrosion resistance coefficient under different numbers of dry-wet cycles.

The flexural strength results of the specimens subjected to different numbers of dry-wet cycles are presented in Table 7. The flexural strength corrosion resistance coefficient of CGC was calculated according to Equation (5), and the calculated results are presented in Table 8. The variation of the flexural strength corrosion resistance coefficient is shown in Figure 4. Under the combined action of sulfate attack and dry-wet cycles, the coefficient first increased and then decreased, reaching its maximum at 30 cycles. In the early stages of attack, cement hydration products and sulfate crystals filled the internal pores of the specimens, enhancing the internal compactness, and the flexural strength was in an increasing phase. As sulfate attack intensified, the sulfate solution gradually crystallized during the drying process, generating crystallization stress. With the increasing number of dry-wet cycles, the accumulation and deposition of sulfate crystals and ettringite caused pore expansion and the formation of microcracks, leading to a decline in flexural strength. The formation of microcracks facilitated the ingress of more SO_4_^2−^ ions into the interior, aggravating the damage. Therefore, as the dry-wet cycle age increased, the flexural strength corrosion resistance coefficient decreased sharply.

#### 2.1.2. Micromorphology Analysis

The mechanical properties and durability of concrete are both closely related to its microstructure. Under the action of sulfate attack and dry-wet cycling, SO_4_^2−^ ions penetrated into the concrete and react chemically with cement hydration products, generating chemical substances that help fill the initial pores and enhance the mechanical properties of CGC [21,22]. To further understand the influence of SO_4_^2−^ ions on the properties of CGC, it was necessary to conduct a micromorphology analysis to reveal the sulfate corrosion mechanism of the concrete.

Under the coupling effect of sulfate attack and dry-wet cycles, the mass loss rate, relative dynamic elastic modulus, and corrosion resistance coefficients of compressive and flexural strength of CGC all presented an upward-then-downward trend. Specifically, the strength corrosion resistance coefficients peaked at 30 cycles, while the mass loss rate and relative dynamic elastic modulus reached their maximum values at 60 cycles. In the early stage, sulfate ions reacted with cement hydration products to generate ettringite and sulfate crystals, which filled internal pores and densified the microstructure, thereby improving the macroscopic performance. After 30 cycles, continuous accumulation of expansive products induced initial microcracks, leading to a slight degradation of strength, whereas the pore-filling effect still dominated until 60 cycles. The 60-cycle condition served as the critical transition point at which the concrete microstructure transformed from densification to damage deterioration. Further increasing the cycles to 90 and 120 only promoted the continuous propagation and connection of microcracks without generating new deterioration mechanisms. Therefore, SEM and XRD analysis were conducted only on specimens after 60 dry-wet cycles. This critical state can effectively reflect the dual characteristics of pore filling and initial cracking, sufficiently revealing the microscopic mechanism responsible for the macroscopic performance evolution of coal gangue concrete. Therefore, this study includes a comparative analysis of the microstructure and variations in the main reaction products of CGC specimens subjected to 0 and 60 sulfate dry-wet cycles.

##### SEM Analysis

**Figure 5:**
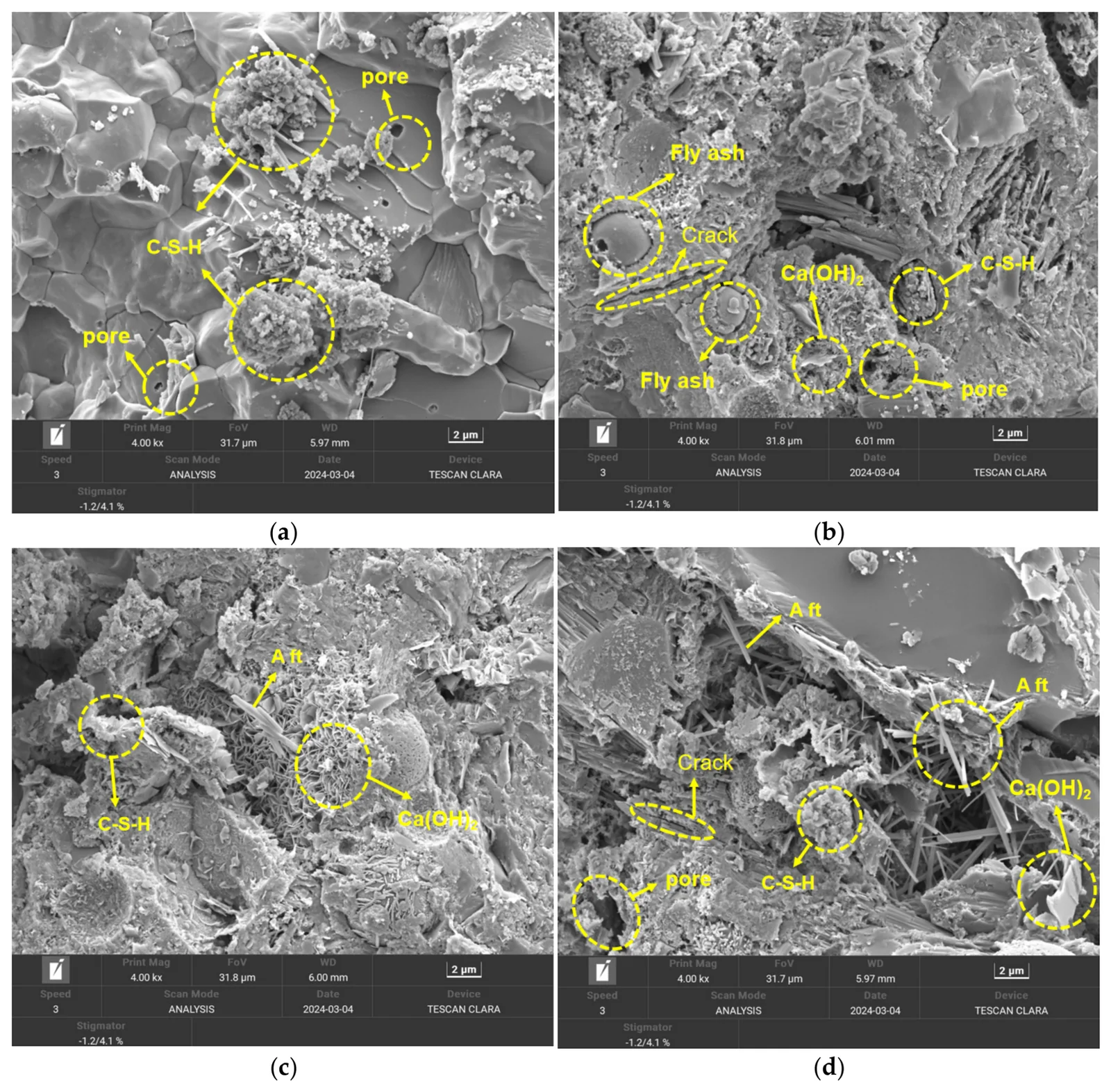
SEM results of specimens with 0 dry-wet cycles. (**a**) distribution of pores and C-S-H gel in specimens; (**b**) coal gangue aggregate, Ca(OH)_2_ crystals, C-S-H gel, and microcracks in specimens; (**c**) coexistence of Ca(OH)_2_, C-S-H gel, and Aft in specimens; (**d**) comprehensive morphology of densely distributed Aft, C-S-H gel, pores, and microcracks in specimens.

To further understand the influence of sulfate on concrete performance, a micromorphology analysis was first conducted on the concrete specimens before sulfate attack (0 dry-wet cycle). As shown in Figure 5a,b, the incorporation of CGC increased the active components Al_2_O_3_ and SiO_2_, which accelerated the pozzolanic reaction. This led to the formation of plate-like Ca(OH)_2_ (CH) crystals. The active components (SiO_2_ and Al_2_O_3_) participated in the secondary hydration reaction of cement, consuming CH crystals and producing ettringite (Aft) and flocculent C-S-H gel, which filled the initial pores inside the specimen. In Figure 5c,d, a small amount of Aft can be observed. With an increasing number of dry-wet cycles, the C-S-H gel and Aft crystals stacked together, forming a uniform and dense structure that filled the pores and voids and improved the internal compactness of the specimen. However, under prolonged sulfate attack, the C-S-H gel is susceptible to decalcification and structural degradation, which gradually weakens the binding matrix.

**Figure 6:**
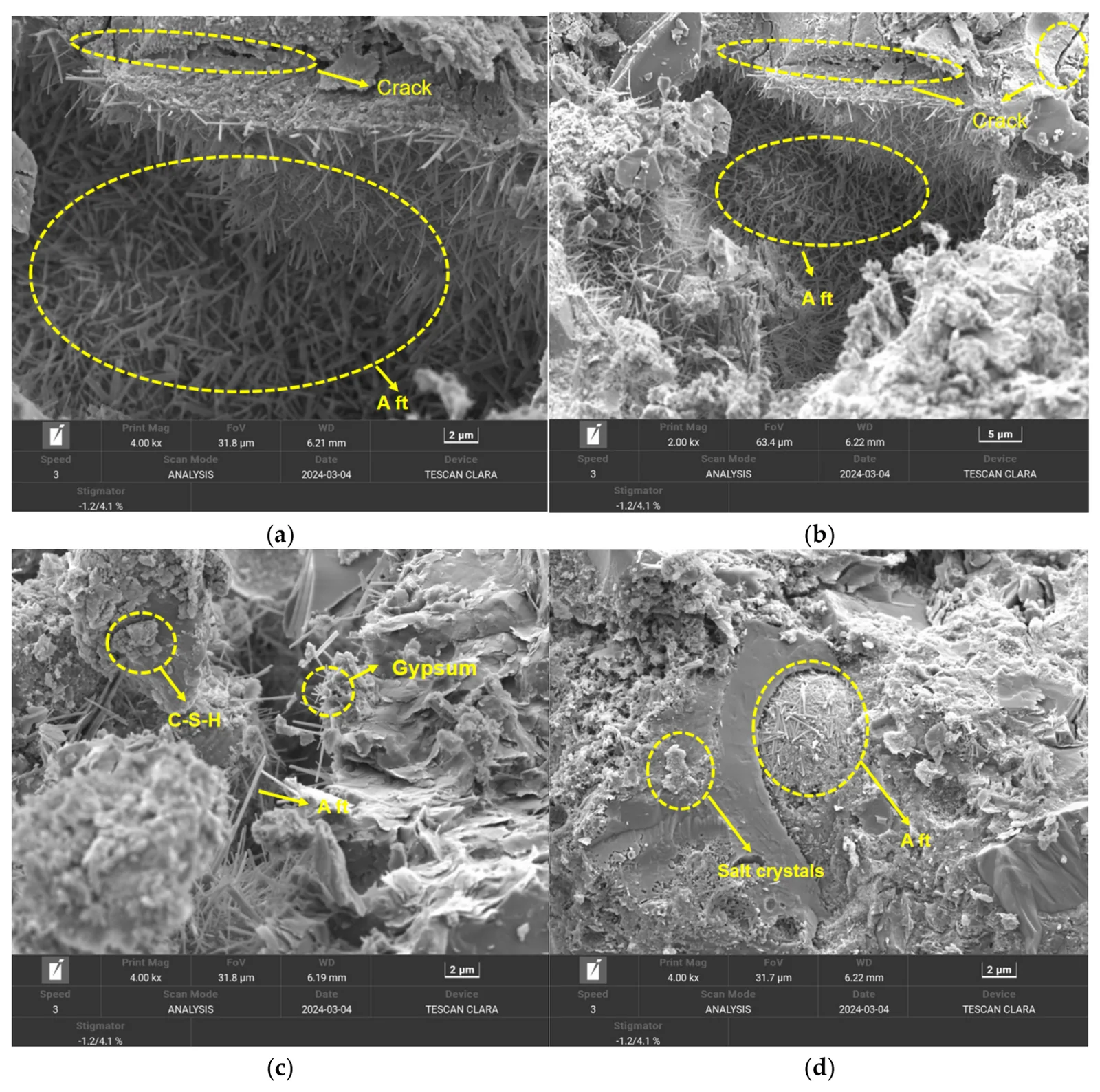
SEM results of the specimen with 60 dry-wet cycles. (**a**) Enrichment of Aft and associated cracks in specimens; (**b**) Morphology of needle-like Aft and microcracks in specimens; (**c**) Characteristics of short columnar gypsum, Aft, residual C-S-H gel, in specimens; (**d**) Morphology of sulfate crystals and Aft attached to aggregate surface in specimens.

Subsequently, a micromorphology analysis was performed on the CGC specimens subjected to 60 dry-wet cycles under sulfate attack. It was found that under sulfate attack, a large amount of SO_4_^2−^ ions penetrated into the interior through the surface pores of the specimen. The cement hydration products reacted with the SO_4_^2−^ ions to generate the expansive product Aft. Although Aft helped fill the internal pores and enhanced the compactness of the specimen, with the increasing number of dry-wet cycles, the Aft content continuously increased and its volume expanded, causing microcracks to form inside the specimen. The progressive propagation of these microcracks led to the loosening, softening, and cracking of the concrete. As shown in Figure 6a,b, a large amount of fine needle-like Aft was observed, accompanied by the appearance of cracks. Figure 6c reveals that SO_4_^2−^ ions from the sulfate environment entered the interior through the pores and reacted with cement hydration products to form short columnar gypsum, along with a small amount of C-S-H gel that had not been entirely consumed by the attack. During the dry-wet cycling process, when the specimen was in a dry state, internal moisture evaporated, increasing the solution concentration and causing sulfate crystallization. Consequently, as can be seen in Figure 6d, small amounts of salt crystals were attached to the aggregate surface.

It is worth noting that all concrete specimens in this study were prepared with a unified cementitious system. The type of hydration products, C-S-H gel microstructure, and phase evolution characteristics of the concrete matrix were basically consistent across all groups, indicating that the differential degradation of mechanical properties was not governed by variations in matrix hydration. Compared with natural aggregate, coal gangue aggregate exhibits inherent differences in surface pore structure and interfacial bonding performance, leading to initial structural defects in the aggregate-paste ITZ of CGC. Under sulfate dry-wet cycling conditions, ITZ with initial defects is more prone to sulfate ion enrichment, ettringite expansion-induced cracking, and sulfate salt crystallization deposition, which further triggers interfacial debonding and progressive microcrack propagation. Accordingly, the varying deterioration degrees of the ITZ are the dominant factor accounting for the differences in sulfate resistance among concrete groups with different coal gangue replacement rates. In this work, the microscopic tests mainly characterized the hydration products and overall microstructure degradation of the concrete matrix. Based on the inherent interfacial characteristics of aggregate, it can be clarified that the differential sulfate deterioration of CGC is primarily controlled by ITZ structural damage rather than changes in the matrix gel system.

##### XRD Analysis

**Figure 7:**
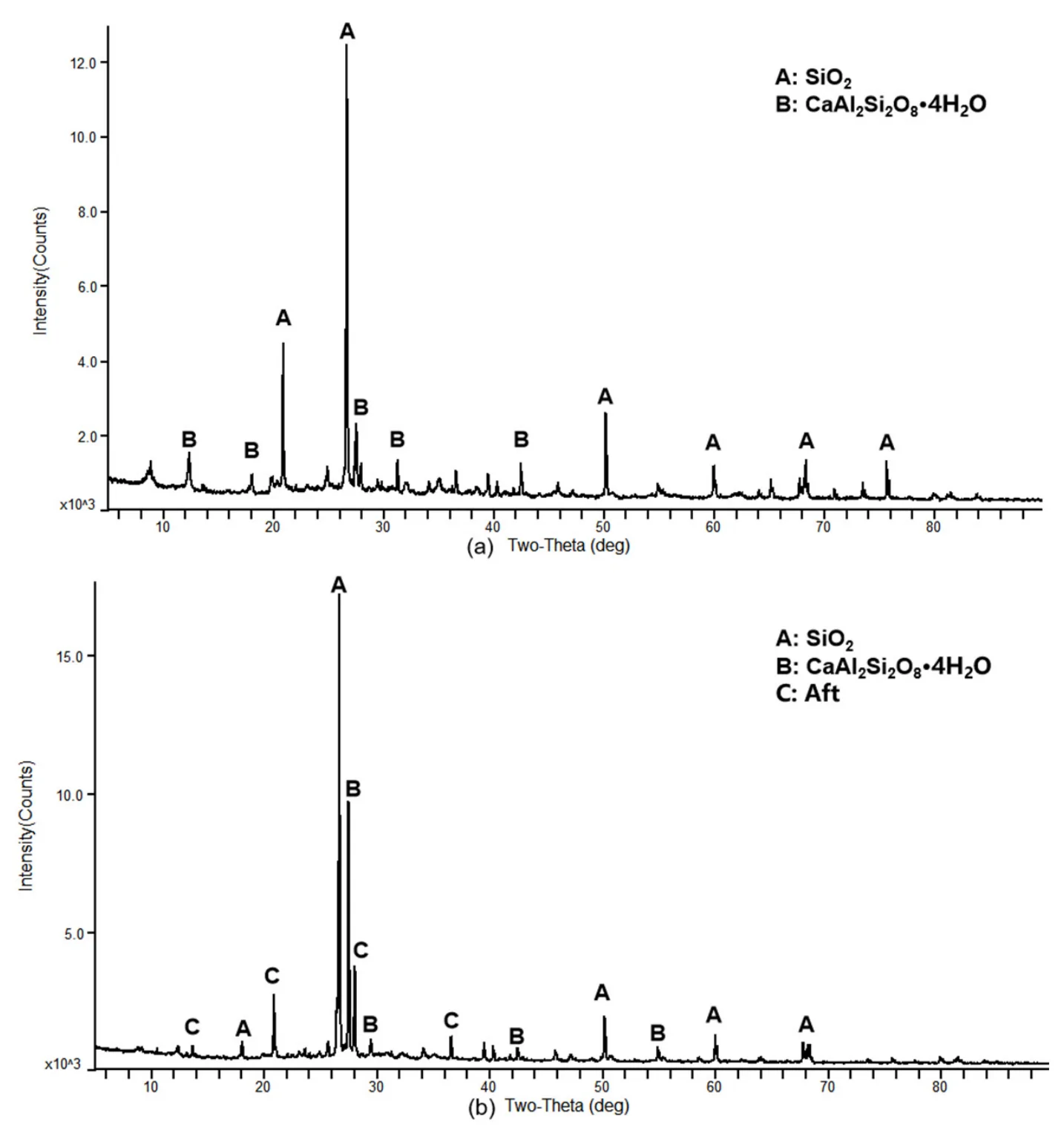
XRD pattern of CGC under sulfate erosion, (**a**) 0 Dry-Wet cycle; (**b**) 60 Dry-Wet cycles.

X-ray diffraction (XRD) analysis was conducted on the CGC specimens before and after sulfate attack, as shown in Figure 7. It can be seen from the figure that before sulfate attack, the Ca(OH)_2_ crystals in the concrete reacted chemically with the active substances (Al_2_O_3_ and SiO_2_) in the coal gangue to form calcium aluminosilicate hydrate (CaAl_2_Si_2_O_8_·4H_2_O). Therefore, SiO_2_ and calcium aluminosilicate hydrate were present in the CGC prior to sulfate attack.

After the CGC was subjected to the coupled action of sulfate and dry-wet cycles, SiO_2_ and calcium aluminosilicate hydrate gradually decreased. This was because the CH crystals in the concrete reacted with the SO_4_^2−^ ions diffusing from the solution to form gypsum, and the calcium aluminosilicate hydrate subsequently reacted with gypsum to generate expansive Aft. In parallel, the C-S-H gel underwent progressive decalcification, leading to a reduction in its Ca/Si ratio and degradation of its binding capacity. At this stage, the diffraction peaks of Aft increased. The Aft produced by sulfate attack and the further hydration of cement formed short columnar gypsum; both of these products filled the internal pores of the concrete, making it denser. As sulfate attack continued, the expansive products Aft and gypsum accumulated progressively, generating expansion stress on the pore walls and creating new cracks and pores. These new cracks provided additional pathways for SO_4_^2−^ ions, accelerating the sulfate attack process.

To semi-quantitatively analyze the relative content variation of hydration products under sulfate attack, a relative intensity ratio method based on XRD raw data was employed, using SiO_2_ as an internal reference. The ratio is defined as follows:

(1) $$R_{i}=\frac{I_{i}}{I_{S{\mathrm{iO}}_{2}}}$$

where, *I*_i_ represents the characteristic diffraction peak intensity of C-S-H gel, Aft, gypsum, or Ca(OH)_2_; $I_{{\mathrm{SiO}}_{2}}$ denotes the peak intensity of quartz at 2θ ≈ 26.6°.

This method is based on the fundamental principle that the integrated intensity of an XRD diffraction peak is positively correlated with the mass fraction of the corresponding phase under identical test conditions. SiO_2_ was chosen as the internal standard because it is chemically inert and remains stable during sulfate attack and dry-wet cycling, thereby minimizing systematic errors caused by sample preparation and instrumental differences. The ratio *R*_i_ can therefore reliably reflect the relative content and evolution of each phase. The semi-quantitative analysis of characteristic peak intensities and relative ratios of main phases before and after sulfate attack are shown in Table 9.

Semi-quantitative analysis based on XRD raw data was conducted to reveal the evolution of gel and erosion products. As shown in Table 9, the relative intensity ratio of C-S-H gel decreased slightly from 0.066 to 0.062 after 60 dry-wet cycles under sulfate attack. This change is mainly attributed to the consumption of part of the C-S-H gel during the sulfate attack process, as well as the massive formation of Aft and gypsum, which reduced the relative proportion of the gel phase.

The characteristic peak of Aft at 2θ ≈ 23.00° increased significantly, indicating that a large amount of Aft was generated with the penetration of sulfate ions. Meanwhile, the characteristic peak intensity of gypsum at 2θ ≈ 20.80° decreased notably, suggesting that part of the gypsum was further consumed to form ettringite, which is consistent with the typical reaction path of sulfate attack in cementitious materials. The characteristic peak intensities of Ca(OH)_2_ remained generally stable, indicating that Ca(OH)_2_ was continuously involved in both the pozzolanic reaction of coal gangue and the sulfate erosion reaction.

Combined with SEM morphological observations, the above semi-quantitative results confirm the microstructural evolution mechanism of coal gangue concrete under sulfate attack and dry-wet cycles. In the early stages, hydration products fill internal pores and densify the matrix; as the number of cycles increases, excessive formation and expansion of ettringite lead to internal stress and microcracking, resulting in structural deterioration.

Based on the combined SEM and XRD results, the chemical changes in the mixture under sulfate attack are primarily manifested as follows: the continuous consumption and structural loosening of C-S-H gel in the original hydration system, a significant reconstruction of the phase proportions within the system, a steady decline in the stable hydrated gel phase, and a progressive increase in expansive erosion phases (ettringite, gypsum) and sulfate crystalline phases. This degradation of the gel structure and the inversion of phase component proportions constitute the fundamental cause of crack propagation and strength loss in concrete.

### 2.2. Prediction Model for the Compressive Strength Corrosion Resistance Coefficient of CGC Under Sulfate Attack

#### 2.2.1. Compressive Strength Corrosion Resistance Coefficient Prediction Model

**Figure 8:**
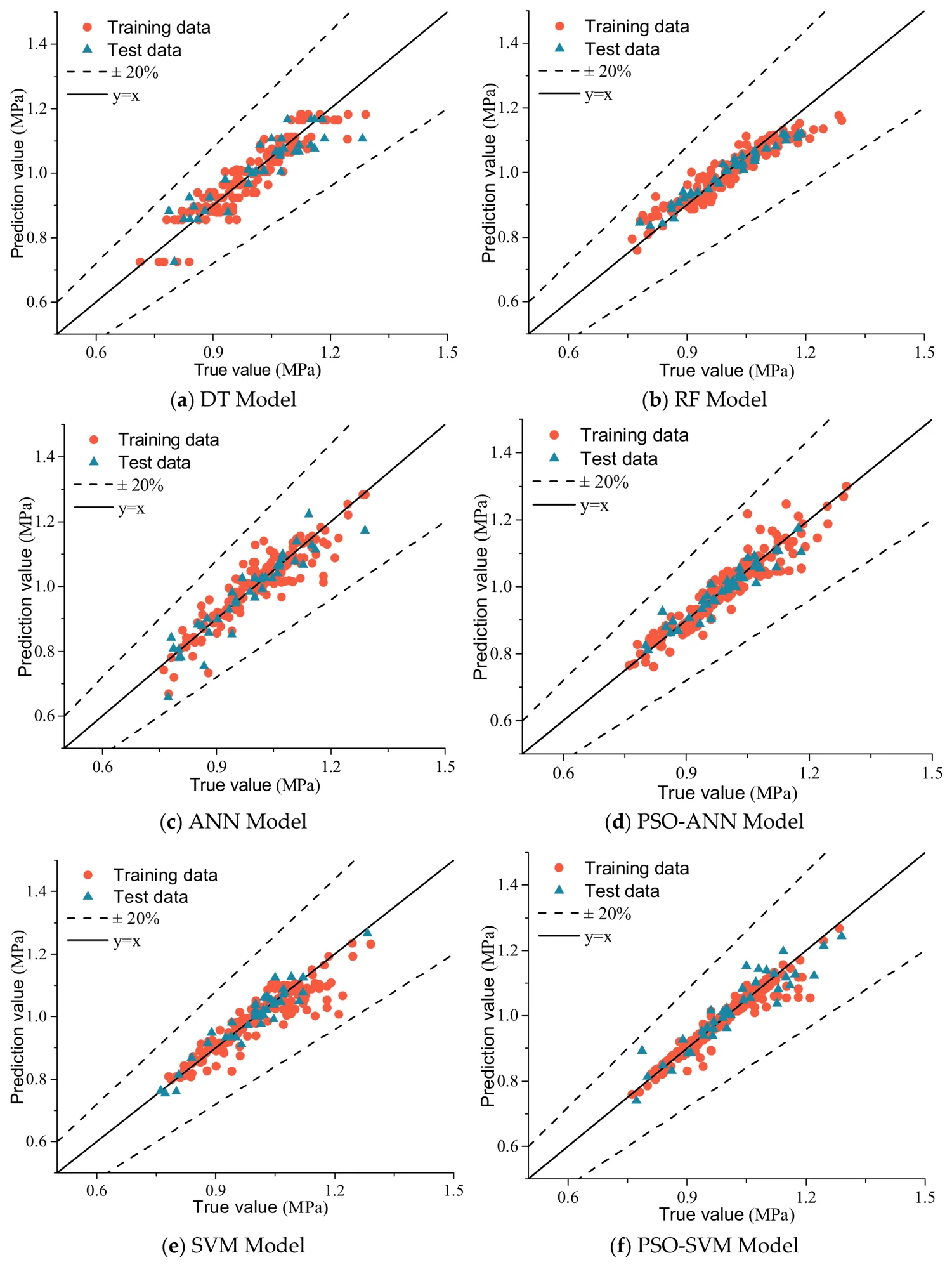
Prediction results of compressive strength corrosion resistance coefficient model.

The data were input into the machine learning models with the hyperparameter values listed in appropriate hyperparameter settings. Six machine learning models for the compressive strength corrosion resistance coefficient were established: decision tree (DT), artificial neural network (ANN), support vector machine (SVM), random forest (RF), particle swarm optimization–artificial neural network (PSO-ANN), and particle swarm optimization–support vector machine (PSO-SVM). Among these, DT, SVM, and ANN are single models, while RF, PSO-ANN, and PSO-SVM are ensemble models. The predicted values were compared with the actual values to characterize the prediction accuracy and fitting capability of the models, as shown in Figure 8. It can be observed that the majority of the data points closely align along the “y = x” line and fall within the ±20% error lines, indicating that all the machine learning models possess satisfactory predictive ability.

In addition, the single models (DT, ANN, SVM) exhibited relatively larger data dispersion during both the training and testing stages. This is because single models have limited generalization ability and are susceptible to data noise, often leading to overfitting, which results in relatively larger data dispersion in the training and testing stages. In contrast, ensemble models (RF, PSO-ANN, PSO-SVM) can reduce the bias and variance of individual models, making them more stable compared to single models, with relatively smaller data dispersion.

The performance of the models was comprehensively evaluated by comparing their prediction accuracy on the training and testing data. The performance metrics of each machine learning model were calculated according to Equations (7)–(11).

The calculated results are presented in Table 10. In the training stage, the R^2^ value of the SVM model was only 0.817. The single SVM model is relatively sensitive to data and parameters, leading to poor data fitting capability and a low R^2^ value. In contrast, the particle swarm optimization algorithm possesses excellent global search capability and can find the global optimum in complex search environments. Therefore, during the training stage, the PSO-SVM model achieved the highest R^2^ value of 0.897. The trained PSO-SVM model also demonstrated high prediction and generalization ability in the testing stage, with an R^2^ value of 0.912.

**Figure 9:**
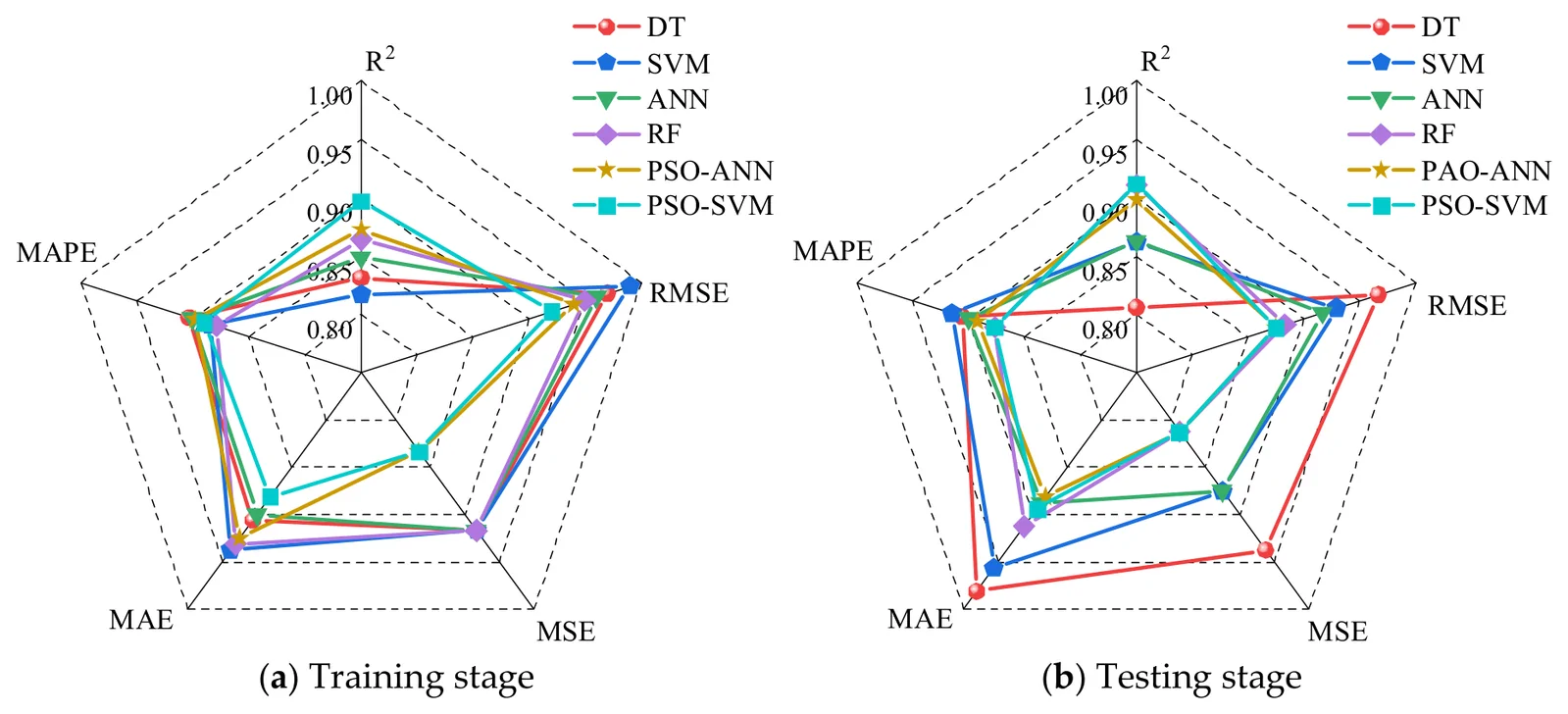
Radar diagram model for compressive strength corrosion resistance coefficient.

Figure 9 presents the evaluation radar chart of the compressive strength corrosion resistance coefficient model. As can be seen from the figure, the ensemble models outperformed the single models in both prediction and generalization ability. This is because an ensemble model is composed of multiple base models, making it more comprehensive and stable than a single model. Even if one learner in the ensemble model makes a biased prediction, the other learners can correct this deviation, thereby improving the model’s prediction and generalization ability.

The trained PSO-SVM model was employed to predict the compressive strength corrosion resistance coefficient of CGC. A total of 20 groups of specimens with varying aggregate replacement rates under sulfate dry-wet cycle erosion were used for model validation in this study. The comparison between the predicted and experimental results is presented in Table 11, which indicates that the proposed PSO-SVM model exhibits excellent prediction accuracy for the corrosion resistance performance of CGC under sulfate erosion conditions.

It should be noted that, although the model was trained on over 200 data points, the observed low sensitivity in the predictions does not stem from insufficient training. Instead, it is primarily attributed to the low variability of the input features. Within the dataset, critical parameters-namely, the water-to-binder ratio, sulfate concentration, and fine aggregate replacement rate-remained essentially constant, causing the training samples to be densely clustered within a confined region of the input space. Under such conditions, the model tends to learn a rather smooth mapping function. Consequently, when applied to the independent set of 20 test samples, the model struggled to produce substantially divergent outputs in response to minor input fluctuations. Macroscopically, this behavior manifests as the observed low prediction sensitivity.

#### 2.2.2. Feature Importance Analysis for the Compressive Strength Corrosion Resistance Coefficient Model

**Figure 10:**
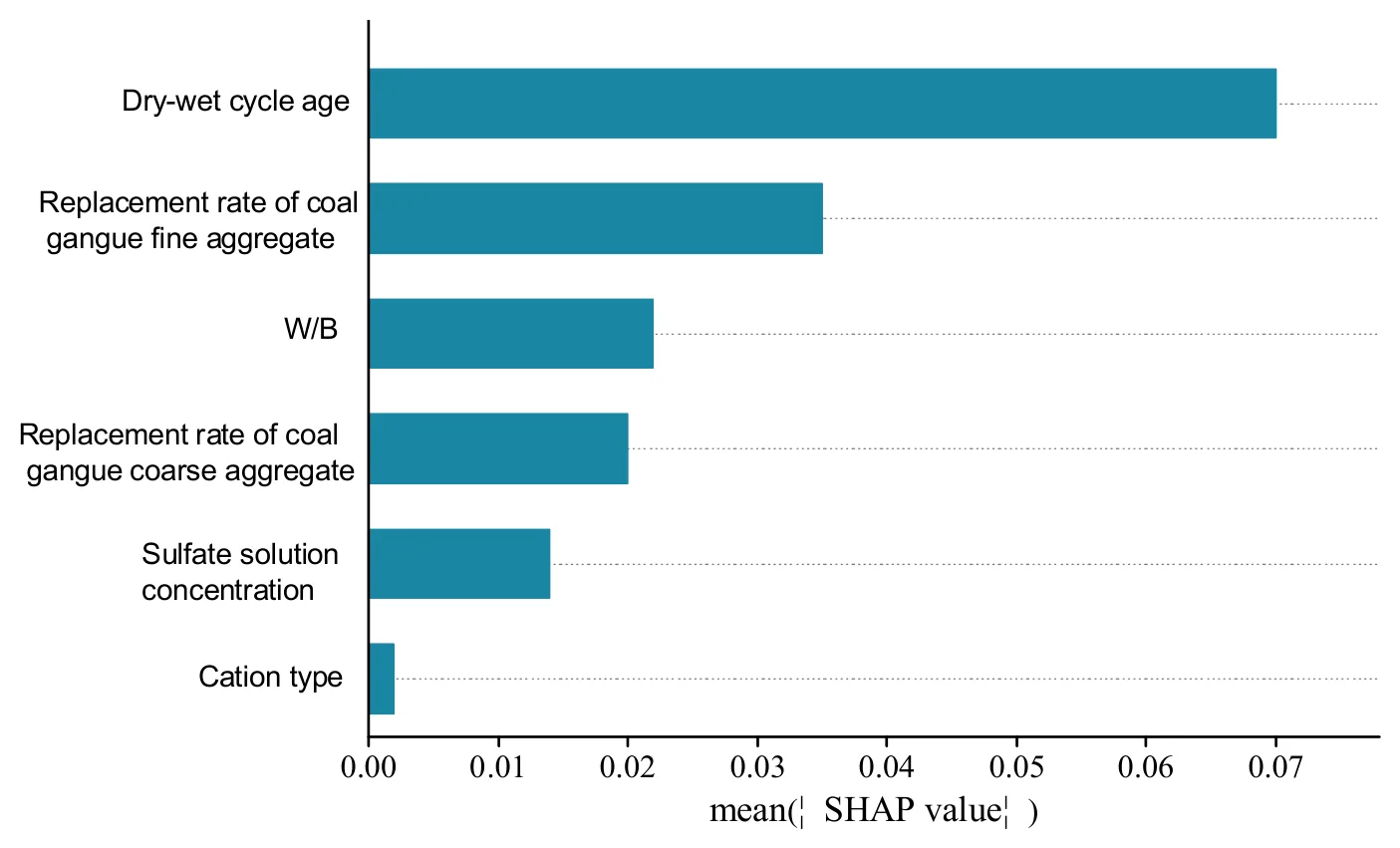
Feature importance analysis for compressive strength corrosion resistance coefficient.

Figure 10 presents the feature importance analysis for the compressive strength corrosion resistance coefficient. The features are ranked in descending order of importance as follows: dry-wet cycle age, coal gangue fine aggregate replacement rate, water-to-binder ratio, coal gangue coarse aggregate replacement rate, sulfate solution concentration, and cation type. Among these, the dry-wet cycle age, coal gangue fine aggregate replacement rate, and water-to-binder ratio exert a relatively large influence on the compressive strength corrosion resistance coefficient of CGC, with SHAP mean absolute values of 0.07, 0.03, and 0.02, respectively. Compared with the three aforementioned influencing factors, the effects of sulfate solution concentration and cation type on the sulfate resistance of CGC are less pronounced.

**Figure 11:**
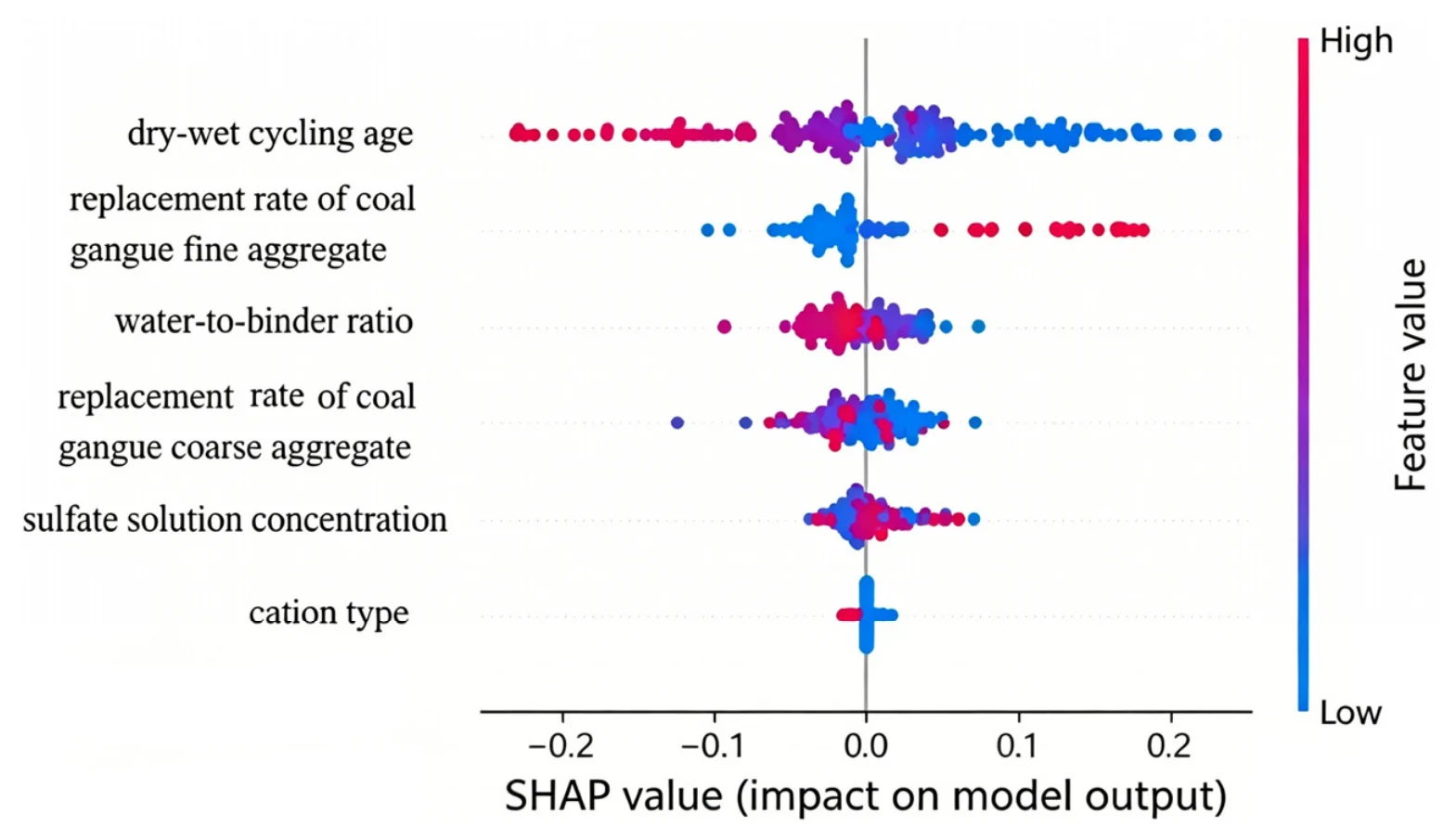
SHAP analysis chart for compressive strength corrosion resistance coefficient.

Figure 11 displays the SHAP summary plot for the compressive strength corrosion resistance coefficient. It can be observed from the figure that the dry-wet cycle age and the water-to-binder ratio exhibit significant negative SHAP values, indicating that both factors have a substantially negative impact on the compressive strength corrosion resistance coefficient. Conversely, the coal gangue fine aggregate replacement rate shows positive SHAP values, indicating that an appropriate replacement rate of coal gangue fine aggregate can, to a certain extent, enhance the compressive strength of CGC under sulfate attack. This is primarily because coal gangue is rich in active substances that promote the pozzolanic effect within the concrete, and the resulting products can fill the internal pores of the CGC, thereby improving its compressive strength.

### 2.3. Prediction Model for the Flexural Strength Corrosion Resistance Coefficient of CGC Under Sulfate Attack

#### 2.3.1. Flexural Strength Corrosion Resistance Coefficient Model

**Figure 12:**
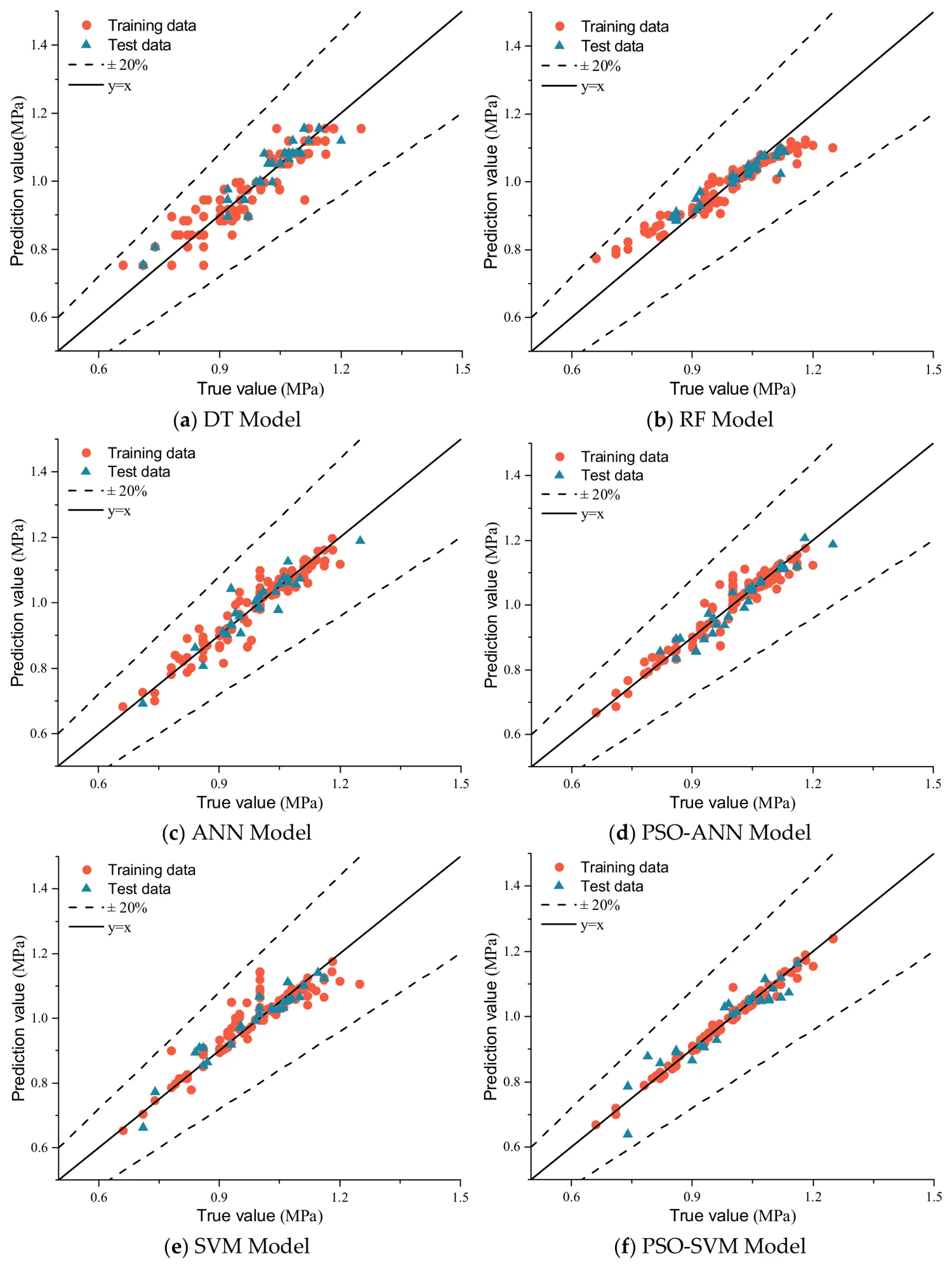
Prediction results of flexural strength corrosion resistance coefficient.

The 133 sets of sample data were randomly divided into training and test sets at a ratio of 4:1. The hyperparameters listed in appropriate hyperparameter settings were then input into three single models (DT, ANN, SVM) and three ensemble models (RF, PSO-ANN, PSO-SVM). The predicted results were compared with the actual values, as shown in Figure 12. Most of the training and test data points are distributed around the line y = x and fall within the ±20% error lines, indicating that all the machine learning models possess good predictive capability. In addition, the training and test data points of the single models exhibit relatively large dispersion, especially for the DT model. In contrast, the data points of the ensemble models closely adhere to the “y = x” line with less scatter, demonstrating that the ensemble models are more stable and have stronger generalization ability and robustness.

The evaluation metrics of the flexural strength corrosion resistance coefficient model were calculated according to Equations (7)–(11), and the results are listed in Table 12. During the training phase, the R^2^ values of the PSO-ANN and PSO-SVM models were 0.911 and 0.924, respectively, revealing a strong capability to capture data features and fit the data, whereas the R^2^ values of the DT and SVM models were relatively low, suggesting a comparatively weaker feature extraction ability. Furthermore, after training, the R^2^ values of the PSO-ANN and PSO-SVM models reached 0.921 and 0.981, respectively, and their RMSE, MAE, and MSE values were relatively smaller than those of the other models. This indicates that the trained PSO-ANN and PSO-SVM models exhibit excellent prediction accuracy and stability.

**Figure 13:**
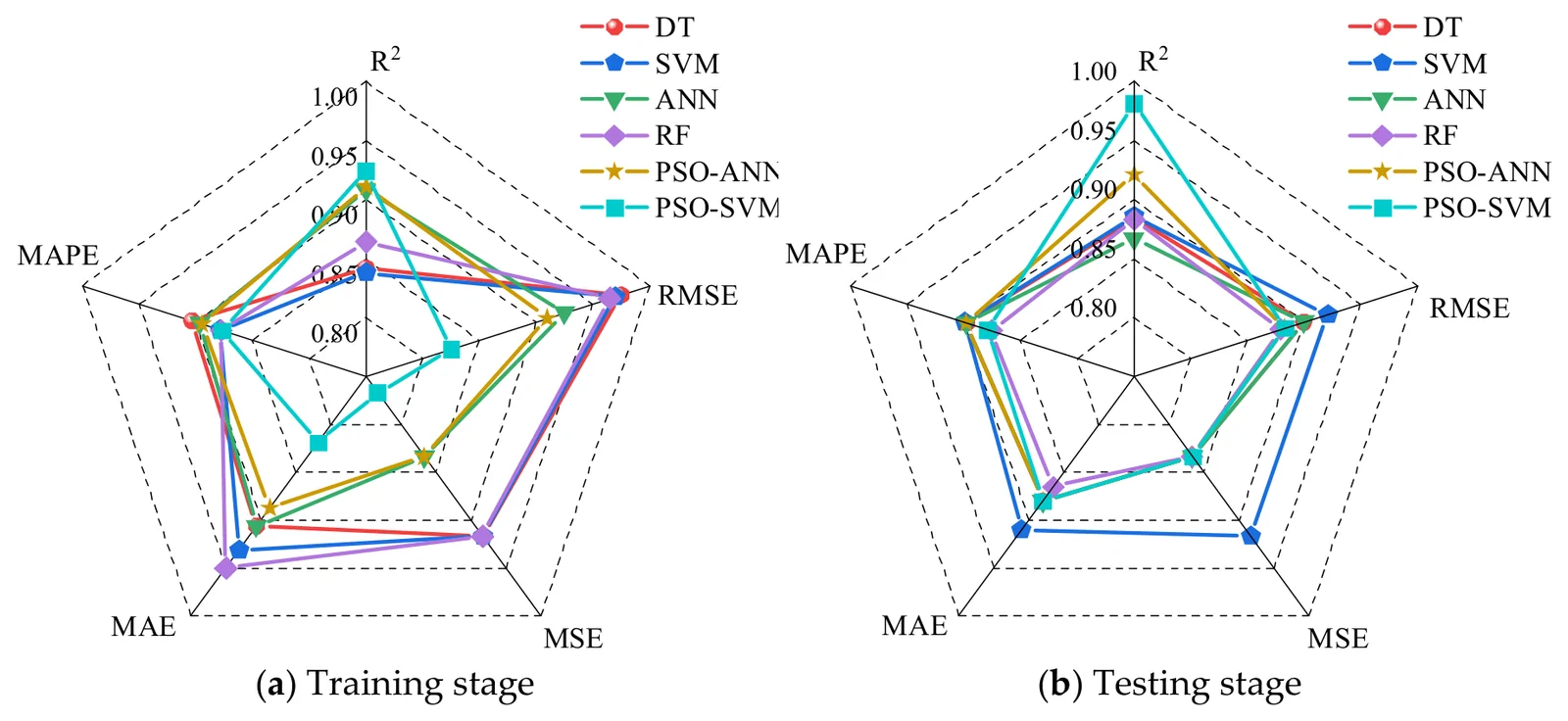
Radar diagram model for flexural strength corrosion resistance coefficient.

Figure 13 presents the radar charts of the evaluation metrics for the six machine learning models. It can be observed that the R^2^ values of the ensemble models in the training phase are all higher than those of the single models, while their MSE, RMSE, MAE, and MAPE values are lower than those of the single models. This is mainly because prediction accuracy tends to improve as model complexity increases. Ensemble models are composed of multiple base models, taking advantage of their strengths to compensate for the weaknesses of individual models. Moreover, ensemble models possess better overall stability and generalization ability. The evaluation metrics of the ensemble models on the test set are superior to those of the single models, but the difference is not substantial, indicating that, compared with single models, the generalization ability of the ensemble models has been improved to a certain degree with the increase in model complexity, though the improvement is limited. Therefore, ensemble models are suitable for predicting the flexural strength corrosion resistance coefficient.

The trained PSO-SVM model was employed to predict the flexural strength corrosion resistance coefficient of CGC. A total of 20 groups of specimens with varying aggregate replacement rates under sulfate dry-wet cycle erosion were used for model validation in this study. The comparison between the predicted and experimental results is presented in Table 13, which indicates that the proposed PSO-SVM model exhibits excellent prediction accuracy for the corrosion resistance performance of CGC under sulfate erosion conditions.

Similar to the case of compressive strength prediction, the relatively low sensitivity observed in the predicted corrosion resistance coefficient of flexural strength can be fundamentally attributed to the low variability of the input features. Since the water-to-binder ratio, sulfate concentration, and fine aggregate replacement rate remained essentially constant across the entire dataset, the training samples were densely clustered within the feature space. Under this constraint, the mapping function learned by the model tends to be rather smooth and is inherently incapable of producing strongly differentiated responses to minor input perturbations. Consequently, on the independent test set, the predicted values of the flexural strength corrosion resistance coefficient likewise exhibit a conservative, low-variance characteristic, consistent with the behavior observed in the compressive strength predictions.

#### 2.3.2. Feature Importance Analysis of the Flexural Strength Corrosion Resistance Coefficient Model

**Figure 14:**
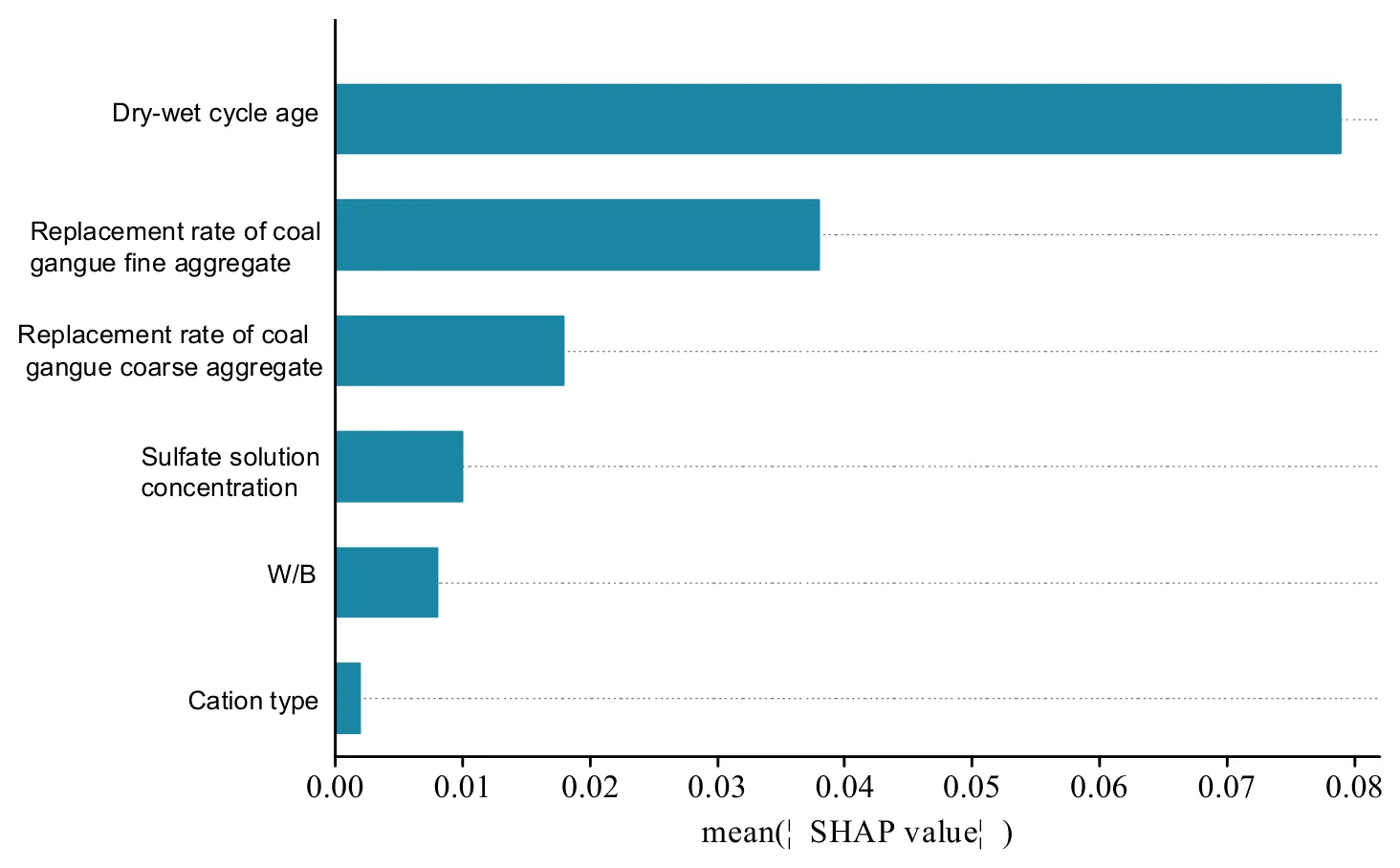
Feature importance analysis for flexural strength corrosion resistance coefficient.

The feature importance analysis of the flexural strength corrosion resistance coefficient is presented in Figure 14. The features are ranked in descending order of importance as follows: dry-wet cycle age, coal gangue fine aggregate replacement rate, coal gangue coarse aggregate replacement rate, sulfate solution concentration, water-to-binder ratio, and cation type. Among these, the dry-wet cycle age yields the highest SHAP mean absolute value of 0.08, indicating that it exerts the most significant influence on the flexural strength corrosion resistance coefficient, followed by the coal gangue fine aggregate replacement rate and the coal gangue coarse aggregate replacement rate, with corresponding SHAP mean absolute values of 0.04 and 0.02, which contribute relatively little to the coefficient.

**Figure 15:**
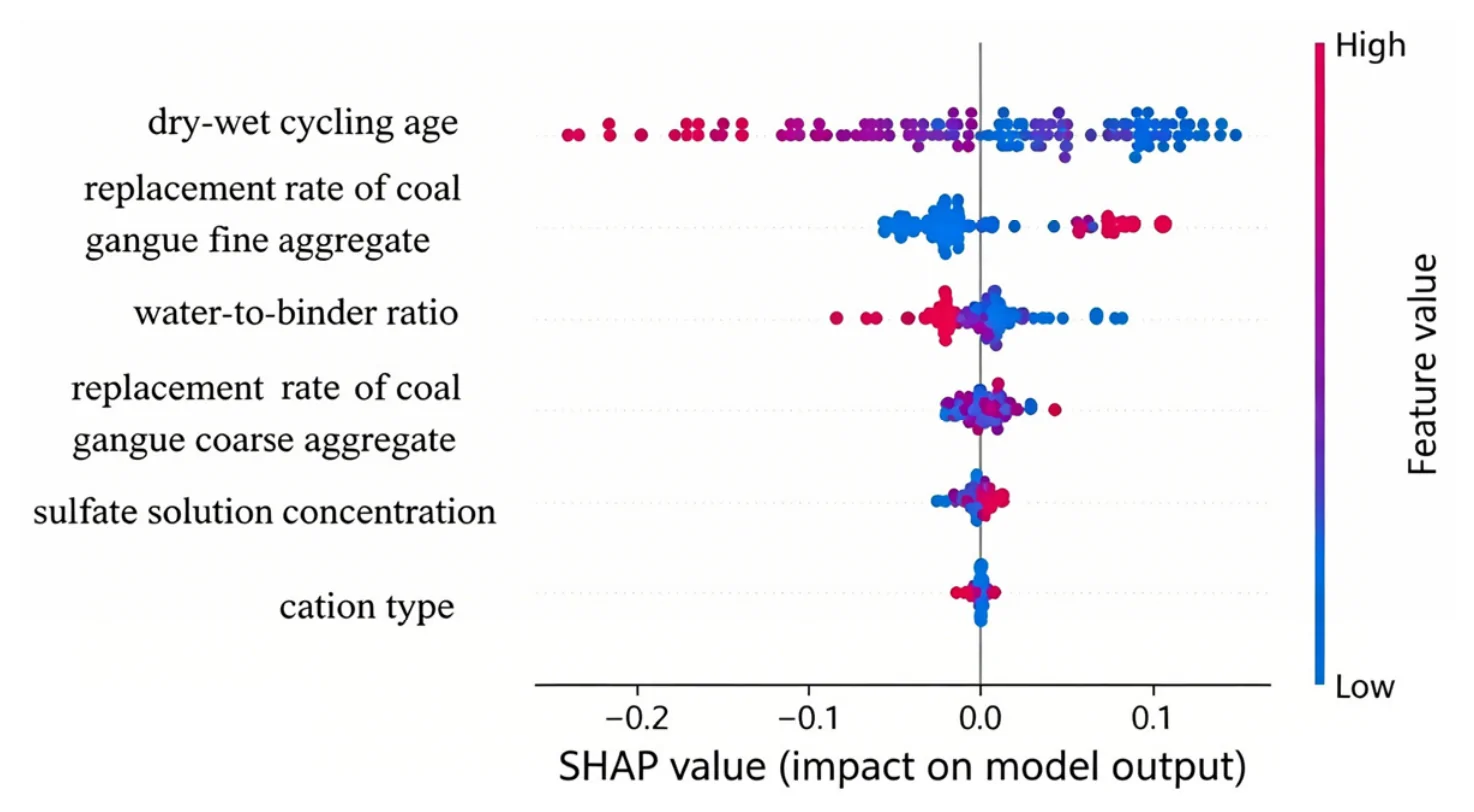
SHAP analysis chart for flexural strength corrosion resistance coefficient.

Figure 15 shows the SHAP summary plot for the flexural strength corrosion resistance coefficient model. It can be observed from the figure that, for the flexural strength corrosion resistance coefficient, the dry-wet cycle age exhibits a strongly negative mean SHAP value, suggesting that an increase in the dry-wet cycle age has a pronounced negative effect on the flexural corrosion resistance coefficient of CGC. In contrast, the coal gangue fine aggregate replacement rate displays a positive mean SHAP value, indicating that raising the content of coal gangue fine aggregate to a certain extent can improve the flexural strength corrosion resistance coefficient of CGC. Meanwhile, the coal gangue coarse aggregate replacement rate samples show a negative mean SHAP value, implying that an excessively high coal gangue coarse aggregate replacement rate adversely affects the flexural strength corrosion resistance coefficient. Therefore, when designing the mix proportion of CGC, an excessively high coal gangue coarse aggregate replacement rate should be avoided, while an appropriate amount of coal gangue fine aggregate can be added to maximize the flexural corrosion resistance coefficient of CGC.

## 3. Conclusions

This study systematically investigated the degradation law and microscopic mechanism of the mechanical properties of CGC through sulfate dry-wet cycling tests, and established an intelligent strength prediction model based on machine learning algorithms. The main conclusions are as follows. 

With an increasing number of dry-wet cycles, the mass loss rate, relative dynamic elastic modulus, as well as compressive and flexural strength corrosion resistance coefficients of CGC exhibited a consistent evolutionary trend of initial growth and subsequent decline. The peak values of compressive and flexural strength corrosion resistance coefficients appeared at 30 dry-wet cycles, while the maximum mass loss rate and relative dynamic elastic modulus occurred at 60 cycles. This staged variation indicates that sulfate dry-wet cycling exerts dual effects on concrete durability; moderate cycling improves the compactness of concrete, whereas prolonged erosion induces severe performance degradation.

Microstructural observations clarified the intrinsic mechanism of the macroscopic performance evolution. After 60 sulfate dry-wet cycles, substantial ettringite and gypsum were generated within the concrete matrix. The early performance improvement is mainly attributed to the pore-filling effect of newly formed hydration and erosion products, which refined the internal pore structure and enhanced matrix compactness. However, continuous accumulation of expansive ettringite together with sulfate crystallization stress produced sustained expansion stress inside the concrete. This stress induced the initiation, propagation, and interconnection of microcracks, destroyed the integrity of the matrix and interfacial structure, and eventually led to the irreversible deterioration of the mechanical properties and durability of the CGC.

The replacement rate of coal gangue coarse aggregate was a decisive factor affecting the sulfate resistance of the CGC, presenting a clear characteristic of low-replacement improvement, moderate-replacement stability, and high-replacement deterioration. Under the condition of fixed 20% fine aggregate replacement, the 20% coarse aggregate replacement group achieved the optimal comprehensive sulfate resistance, because appropriate coal gangue incorporation effectively optimized the pore structure and interfacial transition zone, improved interfacial bonding, and mitigated structural degradation during long-term erosion. The 50% replacement specimen maintained relatively stable durability with minor structural damage and exhibited good adaptability to sulfate dry-wet environments. In contrast, high replacement rates of 80% and 100% introduced numerous initial interfacial defects and connected pores. Sulfate ions easily accumulated at these defective interfaces, promoting excessive formation of expansive products and accelerating microcrack development, thereby causing a sharp drop in corrosion resistance after 60 cycles. Consequently, 20% is identified as the optimal coarse aggregate replacement rate to balance solid waste utilization and sulfate erosion resistance, while excessively high coal gangue coarse aggregate content significantly impaired the microstructure and durability of the concrete, which is inappropriate for sulfate-affected service environments.

Compared with the other models, the PSO-SVM model exhibited superior prediction accuracy and overall stability in predicting both the compressive and flexural strength corrosion resistance coefficients. For the compressive strength corrosion resistance coefficient, the PSO-SVM model achieved R^2^ values of 0.897 and 0.912 in the training and testing phases, respectively; for the flexural strength corrosion resistance coefficient, the corresponding R^2^ values reached 0.924 and 0.981.

Feature importance analysis ranked the influencing factors in descending order of importance. For the compressive strength corrosion resistance coefficient, the order is as follows: dry-wet cycling age > replacement rate of coal gangue fine aggregate > water-to-binder ratio > replacement rate of coal gangue coarse aggregate > sulfate solution concentration > cation type. For the flexural strength corrosion resistance coefficient, the order is as follows: dry-wet cycling age > replacement rate of coal gangue fine aggregate > replacement rate of coal gangue coarse aggregate> sulfate solution concentration > water-to-binder ratio > cation type. Notably, in the feature importance analysis, dry-wet cycling age exhibited a high negative mean SHAP value for both indicators, indicating that dry-wet cycling age exerts a substantial negative impact on both the compressive and flexural strength corrosion resistance coefficients of CGC.

## 4. Materials and Methods

### 4.1. Sulfate Attack Test on CGC

#### 4.1.1. Mix Proportion Design for Sulfate Attack Test

**Figure 16:**
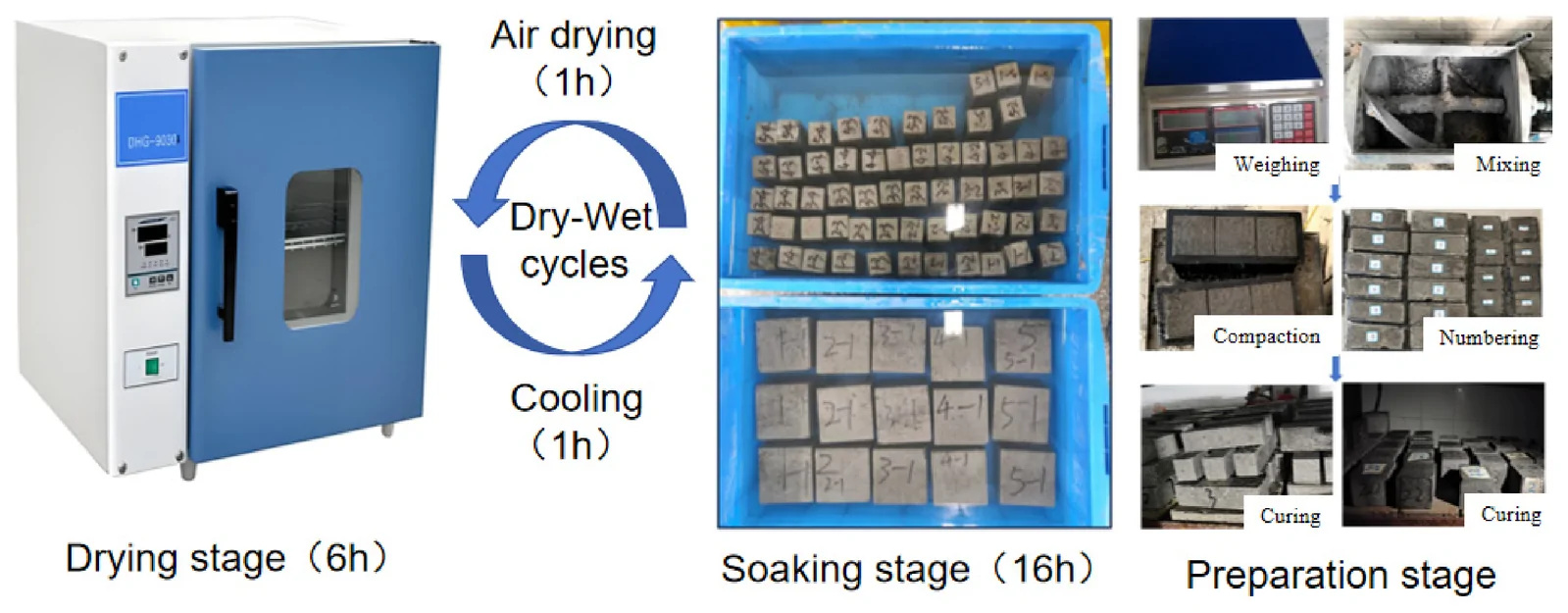
Design of sulfate attack and dry-wet cycle test for CGC.

This study conducted a sulfate attack test on CGC. Based on the mix proportion that exhibited the best performance, the quantities of each component are listed in Table 14. Ordinary Portland cement (P.O 42.5) produced by Xinxiang Tiantai Cement Co., Ltd. (Xinxiang, China) was employed as the cementitious material in this study. The physical properties and chemical compositions of the cement are summarized in Table 15. Grade I fly ash, sourced from Henan Borun Casting Materials Co., Ltd. (Zhengzhou, China), was incorporated at a dosage of 20% by mass as a replacement for cement; its physical properties and chemical composition are presented in Table 16 and Table 17, respectively. Natural crushed stone with a continuous particle gradation of 5–10 mm was used as the coarse aggregate, and its physical properties are listed in Table 18. Natural river sand with a fineness modulus of 2.6 was used as the fine aggregate, with an apparent density of 2648 kg/m^3^ and a bulk density of 1470 kg/m^3^. The coal gangue coarse and fine aggregates were both obtained from the Hebi coal mining area in Henan Province. The coal gangue coarse aggregate had a continuous particle gradation of 5–10 mm, and the coal gangue fine aggregate had a continuous particle gradation of 0–5 mm. The physical properties of the coal gangue coarse and fine aggregates used in the tests are presented in Table 19. A polycarboxylate superplasticizer produced by Shanxi Feike New Material Technology Co., Ltd. (Yuncheng, China), with a water reduction rate of up to 25%, was used. Magnesium sulfate (MgSO_4_) was obtained from Tianjin Zhiyuan Chemical Reagent Co., Ltd. (Tianjin, China), and a 10% MgSO_4_ solution was used as the aggressive medium. Four dry-wet cycle numbers were designed, i.e., 30, 60, 90, and 120 cycles. Full immersion was adopted, with the solution level maintained at 30 mm above the top surface of the specimens. The dry-wet cycle process is illustrated in Figure 16. CGC specimens were prepared with a fixed fine aggregate replacement rate of 20% and coarse aggregate replacement rates of 0, 20%, 50%, 80%, and 100%, respectively. For each mix proportion, 60 cubic specimens with dimensions of 100 mm × 100 mm × 100 mm and 60 prismatic specimens with dimensions of 40 mm × 40 mm × 160 mm were cast. All specimens were subjected to dry-wet cycles in a 10% MgSO_4_ solution, with the number of cycles set at 30, 60, 90, and 120. Upon reaching the designated cycles, the cubic specimens were tested for mass loss rate, relative dynamic modulus of elasticity, and the corrosion resistance coefficient of compressive strength, while the prismatic specimens were evaluated for the corrosion resistance coefficient of flexural strength. The design of the sulfate attack specimens is presented in Table 20.

#### 4.1.2. Test Methods

The mass, dynamic elastic modulus, compressive strength, and flexural strength of the CGC specimens after sulfate attack were tested and analyzed. The compressive and flexural strength tests were carried out in accordance with the Standard for Test Methods of Physical and Mechanical Properties of Concrete (GB/T 50081-2019) and the Test Code for Hydraulic Concrete (SL 352-2020) [23,24].

1. Mass test method

In accordance with the Standard for Test Methods of Long-term Performance and Durability of Ordinary Concrete (GB/T 50082-2009) [25], the mass loss rate of specimens that reached the specified number of dry-wet cycles was calculated according to Equation (2):

(2) $$M=\frac{m_{n}-m_{1}}{m_{1}}\times 100\%$$

where m_1_ is the mass of the specimen before dry-wet cycle test (g); m_n_ is the mass of the specimen after n dry-wet cycle test (g); M is the mass loss rate of the specimen after n dry-wet cycles (%).

2. Dynamic elastic modulus test method

**Figure 17:**
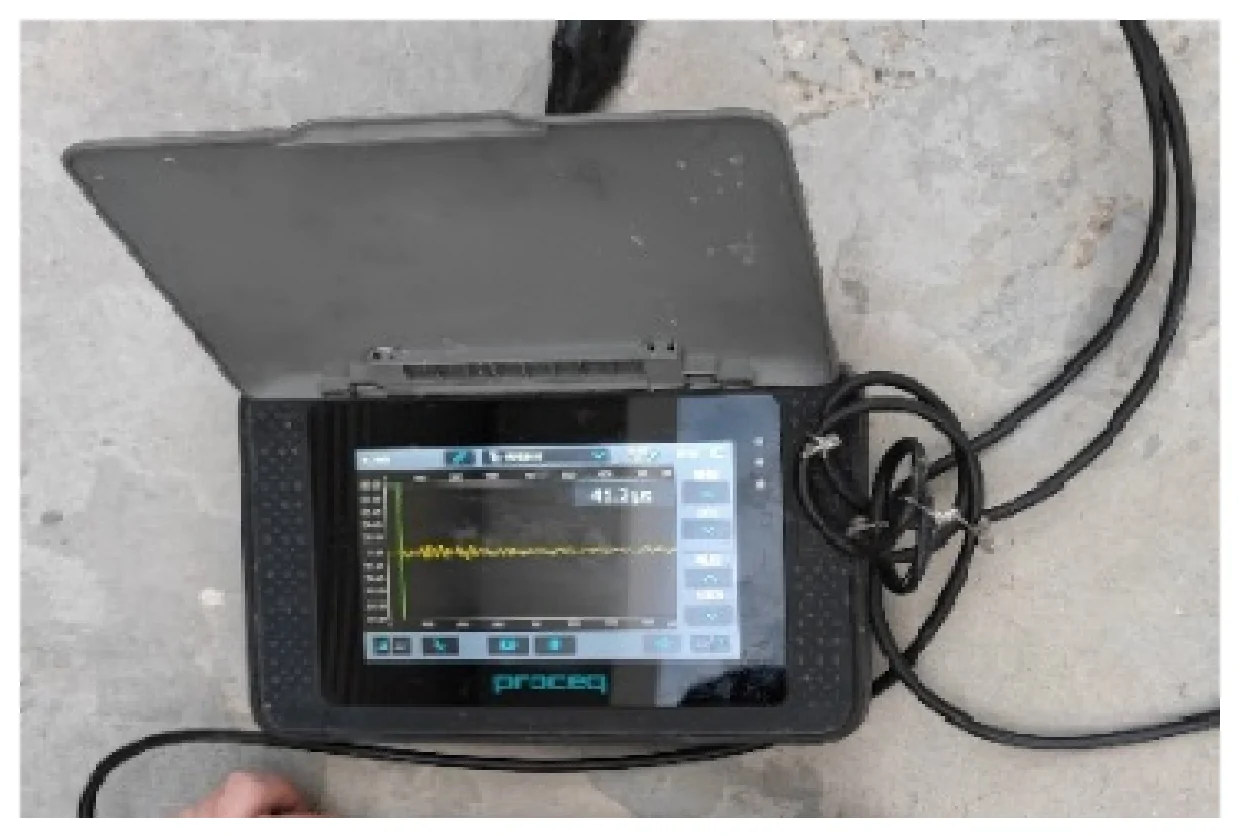
Ultrasonic nondestructive tester.

Before and after sulfate solution attack, the transit time of the concrete specimens was measured using an ultrasonic nondestructive tester, as shown in Figure 17. The dynamic elastic modulus of the concrete specimens was calculated according to Equation (3), and the relative dynamic elastic modulus was calculated according to Equation (4) as follows:

(3) $$E_{d}=\frac{\left(1+\nu \right)\left(1-2\nu \right)\rho V^{2}}{\left(1-\nu \right)}=\frac{\left(1+\nu \right)\left(1-2\nu \right)\rho L^{2}}{\left(1-\nu \right)t^{2}}$$

(4) $$E_{\mathrm{rd}}=\frac{E_{n}}{E_{0}}=\frac{V_{n}^{2}}{V_{0}^{2}}=\frac{t_{0}^{2}}{t_{n}^{2}}$$

where V is the ultrasonic wave velocity (m/s); ρ is the density of the specimen (kg/m^3^); ν is Poisson’s ratio; E_d_ is the dynamic elastic modulus of the specimen (MPa); t is the ultrasonic transit time of the specimen (μs); E_n_ is the dynamic elastic modulus of the specimen after n dry-wet cycles; E_0_ is the dynamic elastic modulus of the specimen at 0 cycle; V_n_ is the ultrasonic wave velocity after n dry-wet cycles (m/s); V_0_ is the ultrasonic wave velocity at 0 dry-wet cycle (m/s); t_0_ is the ultrasonic transit time of the specimen at 0 dry-wet cycle (μs); t_n_ is the ultrasonic transit time of the specimen after n dry-wet cycles (μs); E_rd_ is the relative dynamic elastic modulus of the specimen after n dry-wet cycles.

3. Compressive strength corrosion resistance coefficient

Since the sulfate attack test on CGC is influenced by multiple factors, this paper chooses to use the “compressive strength corrosion resistance coefficient” as an indicator to evaluate the resistance of CGC to sulfate attack. In accordance with the Standard for Test Methods of Long-term Performance and Durability of Ordinary Concrete (GB/T 50082-2009) [25], the formula for the compressive strength corrosion resistance coefficient of coal gangue aggregate concrete under sulfate attack is given by Equation (5), as follows:

(5) $$K_{c}=\frac{f_{n}}{f_{1}}\times 100\%$$

where K_c_ is the compressive strength corrosion resistance coefficient (%); f_n_ is the compressive strength value of the sulfate-attacked concrete specimen after n dry-wet cycles (MPa); f_1_ is the compressive strength value of the standard-cured concrete specimen at the same age as the sulfate-attacked specimen (MPa).

4. Flexural strength corrosion resistance coefficient

The flexural strength corrosion resistance coefficient is calculated according to Equation (6) [25], as follows:

(6) $$K_{f}=\frac{f_{fn}}{f_{f0}}\times 100\%$$

where K_f_ is the flexural strength corrosion resistance coefficient (%); f_fn_ is the flexural strength value of the sulfate-attacked concrete specimen after dry-wet cycle testing (MPa); f_f0_ is the flexural strength value of the standard-cured concrete specimen at the same age as the sulfate-attacked specimen (MPa).

5. Microstructure and phase analysis

To reveal the micro-deterioration mechanism of CGC under the coupling effect of sulfate attack and dry-wet cycles and clarify the micro-essence of the macroscopic mechanical property evolution, SEM microscopic morphology observation and XRD phase analysis were conducted on both uneroded and eroded CGC specimens in this study. Concrete specimens with different coal gangue coarse aggregate replacement rates exhibit consistent macroscopic performance evolution trends and highly similar micro-corrosion product types and deterioration mechanisms under sulfate erosion conditions. To avoid accidental errors caused by a single mix proportion and objectively reflect the typical micro-corrosion characteristics of CGC under corresponding erosion conditions, micro-test samples were collected from specimens with different coarse aggregate replacement rates subjected to the same number of dry-wet cycles.

All micro-samples were taken from the intact core regions of specimens after mechanical property tests, while surface layers, cracks and severely damaged areas were discarded to eliminate the interference of surface carbonation, direct solution scouring, and external mechanical damage [11,12]. The screened samples from different mix proportions were uniformly mixed to comprehensively characterize the microstructure morphology and hydration product evolution of CGC under sulfate erosion, improving the representativeness and reliability of micro-test results. After sampling, the specimens were immediately immersed in anhydrous ethanol to terminate cement hydration and then vacuum-dried for subsequent testing. SEM was adopted to observe the pore structure, interface morphology and microscopic damage characteristics, and XRD was used to analyze the variations of hydration products and corrosion mineral phases before and after erosion. On this basis, the micro-deterioration mechanism of CGC under sulfate dry-wet cycles was systematically clarified.

### 4.2. Machine Learning Model

Constructing a machine learning predictive model mainly involves three steps: data collection, model building, and model performance evaluation. A basic model is called a single model, whereas an ensemble model combines multiple single base models. However, a single machine learning model often struggles to maintain high prediction accuracy [26]. This paper compares several optimization algorithms for machine learning models and their performance evaluation metrics.

Artificial neural networks (ANNs) are mathematical models that mimic the biological neural networks of the human brain, possessing powerful nonlinear mapping capabilities. An ANN mainly consists of an input layer, hidden layers, and an output layer. The input layer is primarily responsible for receiving data, while the output layer delivers the information processing results. The hidden layer is composed of a large number of nodes (neurons) and is mainly used for information propagation and extracting relevant features from the input data, analogous to the synapses in neural structures [27]. In an ANN, the input layer receives sample data; then, each neuron computes a weighted sum of the input data according to the assigned weights, applies an activation function, and propagates the result to the output layer to generate the output.

A support vector machine (SVM), as a single machine learning algorithm, classifies data features and outputs predicted values by developing an optimal hyperplane in a multidimensional space. In an SVM, the data points closest to the hyperplane are called support vectors.

A decision tree (DT) model is composed of a root node, internal nodes, and leaf nodes. Constructing a decision tree model involves four aspects: processing the raw data, generating the DT, pruning, and performance evaluation. The DT begins partitioning from the original node, making a decision and outputting a result at each leaf node it passes through. The generation of each new node represents the completion of a classification. After a leaf node completes the decision of the current original node, it becomes the root node of the next tree level to transmit information, and the process repeats in this manner until no further splitting is possible. The root node and leaf nodes are interconnected to form a decision tree [28].

Random forest (RF), as an ensemble algorithm, consists of multiple decision trees, each of which performs a small tree-based regression task. The main principle of random forest is to randomly draw n samples from the original dataset N for training to form a new training set. In this way, multiple decision trees are constructed and combined to create the random forest [29,30]. Random forest builds multiple decision tree models through random sampling and random feature selection.

Particle swarm optimization (PSO) is a bio-inspired algorithm. Its fundamental principle is based on the observation of bird flocking behavior. Through cooperation and information sharing among individuals within the population, the behavior of the entire swarm evolves from disorder to order, ultimately finding the optimal path.

Data normalization is one of the critical steps in machine learning. If the ranges of the input data differ, it becomes difficult for a machine learning model to make effective and accurate predictions. Data normalization adjusts all input variables to the same order of magnitude, which is accomplished using a min-max mapping function, as shown in the following Equation (7) [31]:

(7) $$X_{n}=\frac{X-X_{\min}}{X_{\max}-X_{\min}}$$

where $X_{n}$ is the normalized data, $X_{\min}$ and $X_{\max}$ are the minimum and maximum values of each input variable, and X is the sample data. Data normalization can speed up computation while improving the accuracy and stability of the predictive model. Therefore, this study randomly split the sample data into training and testing sets at a ratio of 4:1.

This paper uses root mean square error (RMSE), mean square error (MSE), mean absolute error (MAE), mean absolute percentage error (MAPE), and coefficient of determination (R^2^) as the evaluation metrics for the machine learning model. The specific formulas are as follows [31,32,33]:

(8) $$\mathrm{RMSE}=\sqrt{\frac{1}{n}\sum_{i=1}^{n}{\left(M^{\prime }-M\right)}^{2}}$$

(9) $$\mathrm{MSE}=\frac{1}{n}{\sum_{i=1}^{n}\left(M^{\prime }-M\right)}^{2}$$

(10) $$\mathrm{MAE}=\frac{1}{n}\sum_{i=1}^{n}\left|M-M^{\prime }\right|$$

(11) $$\mathrm{MAPE}\left(\%\right)=\frac{1}{n}\sum_{i=1}^{n}\left|\frac{M^{\prime }-M}{M}\right|\times 100$$

(12) $$R^{2}=1-\frac{\sum_{i=1}^{n}{\left(M-M^{\prime }\right)}^{2}}{\sum_{i=1}^{n}{\left(M-\bar{M}\right)}^{2}}$$

where $M^{\prime }$ is the predicted value, M is the actual value, $\bar{M}$ is the mean of the actual values, and *n* is the number of samples. The values of MSE, RMSE, MAE, and MAPE are inversely proportional to the prediction accuracy of the model; that is, the lower the values of MSE, RMSE, MAE, and MAPE, the higher the prediction accuracy, and vice versa. In addition, the closer the coefficient of determination R^2^ is to 1, the higher the accuracy of the model. These evaluation metrics reflect the prediction accuracy of the model by comparing the differences between the actual and predicted values.

#### 4.2.1. Prediction Model for the Compressive Strength Corrosion Resistance Coefficient of CGC Under Sulfate Attack

##### Data Collection and Analysis for the Compressive Strength Corrosion Resistance Coefficient Model

By combining the experimental data obtained in this study with data extracted from six published references [21,34,35,36,37,38], a total of 204 datasets related to the compressive strength corrosion resistance coefficient of CGC under sulfate attack were collected. Table 21 provides a statistical description of these data, listing the data ranges of the input and output features for the prediction model.

**Figure 18:**
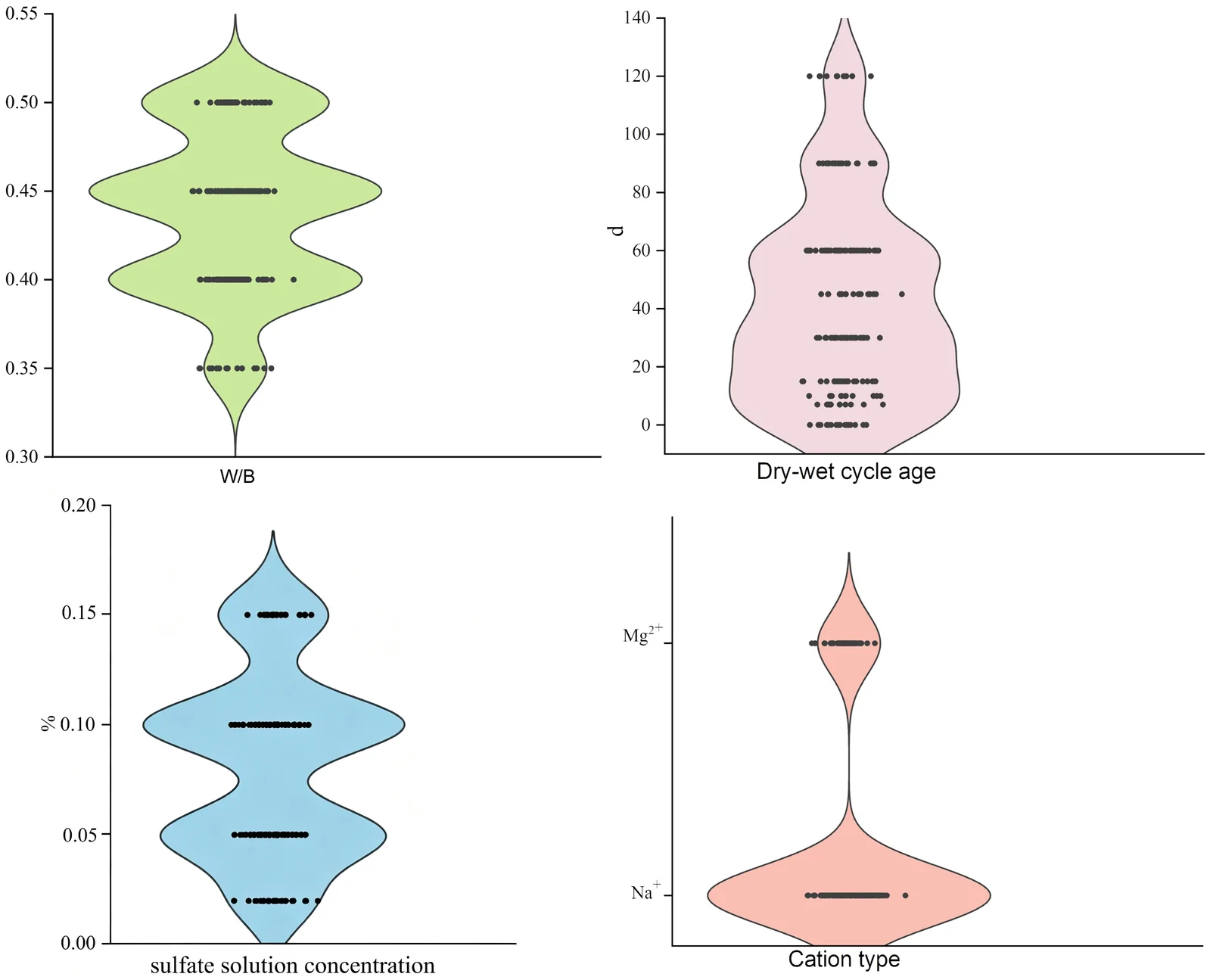

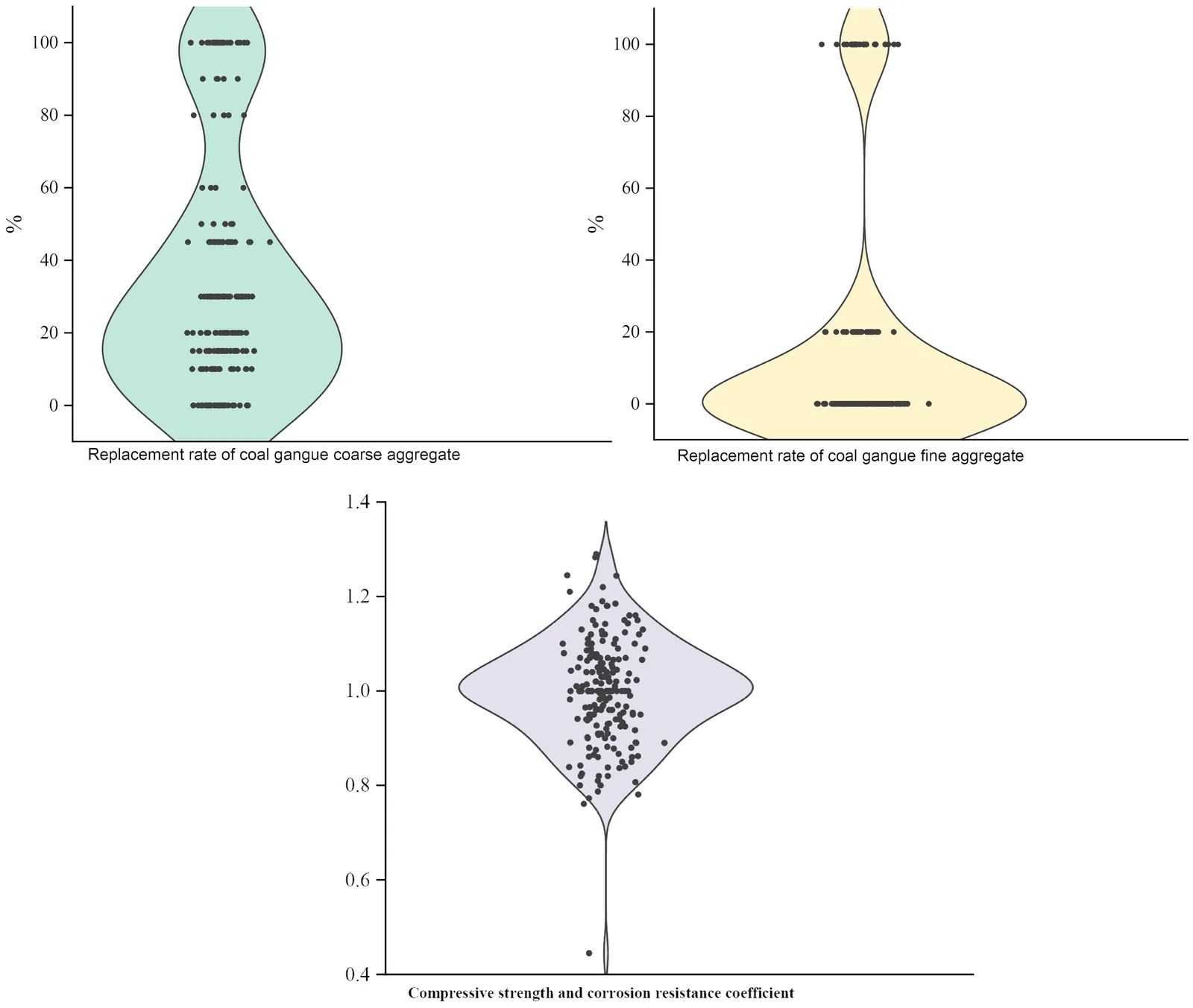
Data distribution diagram of compressive strength corrosion resistance coefficient model.

The input features include the water-to-binder ratio, dry-wet cycle age, sulfate solution concentration, coal gangue coarse aggregate replacement rate, coal gangue fine aggregate replacement rate, and cation type, totaling six input features. The output feature is the compressive strength corrosion resistance coefficient. The data distribution of the input and output features is shown in Figure 18.

**Figure 19:**
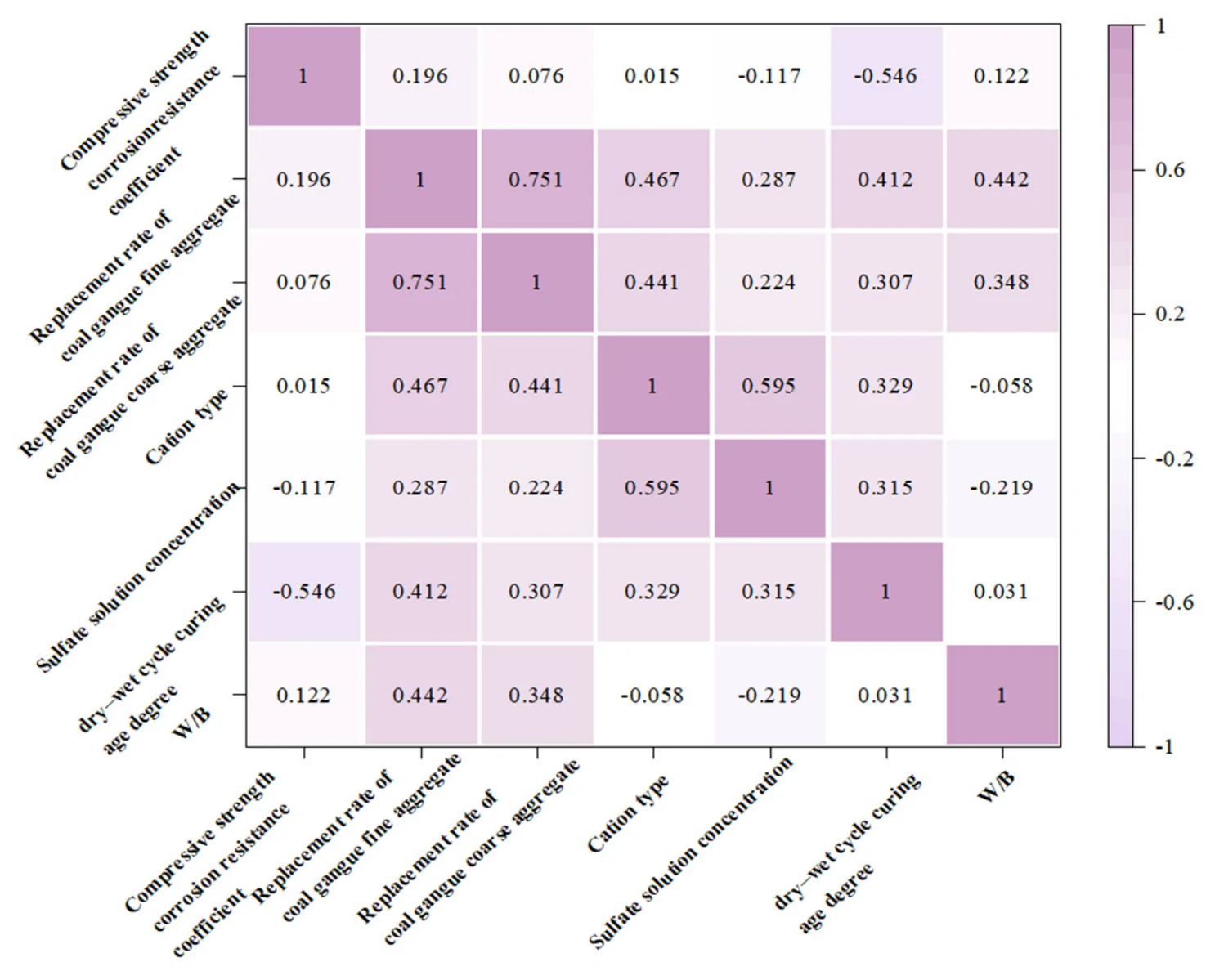
Pearson correlation diagram of compressive strength corrosion resistance coefficient model.

Figure 19 presents the Pearson correlation coefficient matrix between the input and output features of the model. As can be seen from the figure, the dry-wet cycle age and the sulfate solution concentration exhibit a significant negative correlation with the compressive strength corrosion resistance coefficient, with correlation coefficients of –0.546 and –0.117, respectively, indicating that both factors have a negative impact on the coefficient. In contrast, the coal gangue fine aggregate replacement rate and the water-to-binder ratio show a positive correlation with the compressive strength corrosion resistance coefficient, with positive correlation coefficients of 0.196 and 0.122, respectively, suggesting that these two factors exert a positive influence on the coefficient.

##### Determination of Hyperparameters for the Compressive Strength Corrosion Resistance Coefficient Model

Each machine learning model possesses multiple hyperparameters, and different choices of hyperparameters can lead to different prediction results. Therefore, it is necessary to optimize both the number and the types of hyperparameters to achieve the optimal performance of the machine learning model [39]. In this study, an iterative approach was adopted to optimize the hyperparameters of the machine learning models. Each model underwent multiple tuning iterations until the error was minimized and became stable. The determined hyperparameter values for the compressive strength corrosion resistance coefficient model are presented in Table 22.

#### 4.2.2. Prediction Model for the Flexural Strength Corrosion Resistance Coefficient of CGC Under Sulfate Attack

##### Data Collection and Analysis for the Flexural Strength Corrosion Resistance Coefficient Model

**Figure 20:**
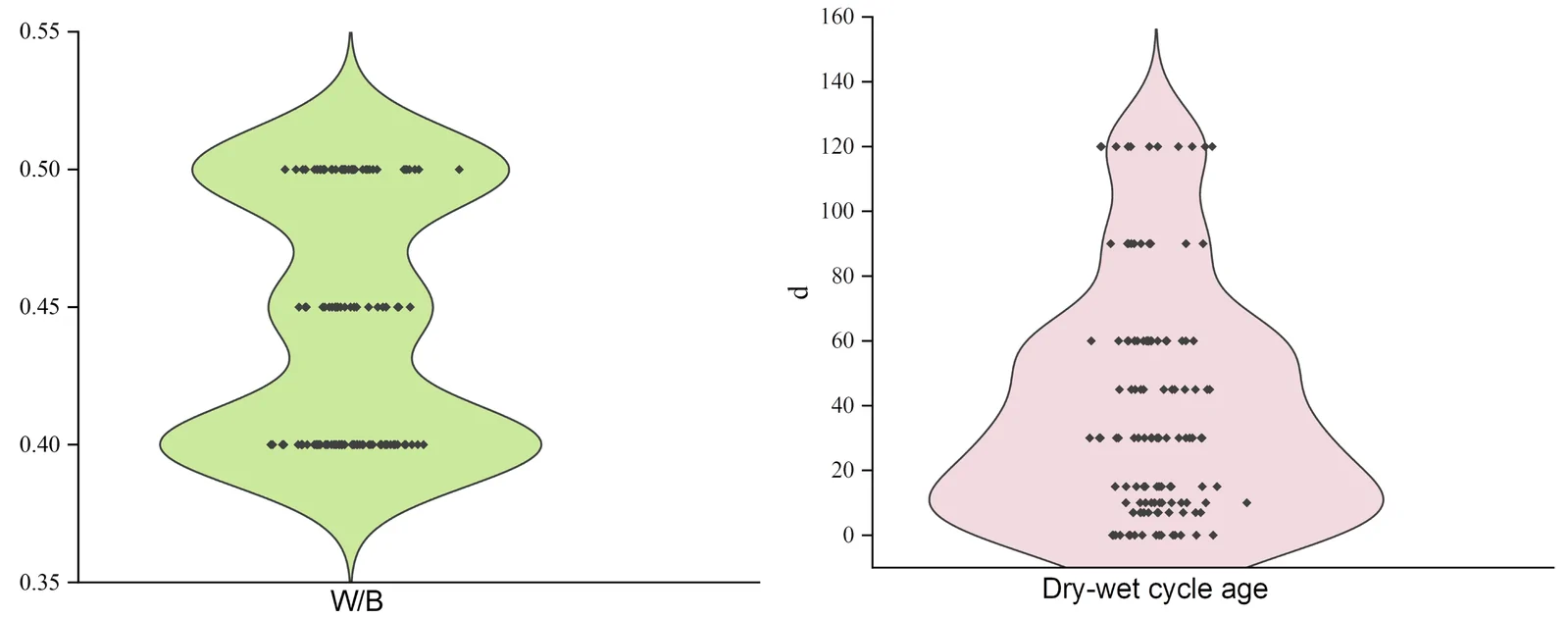

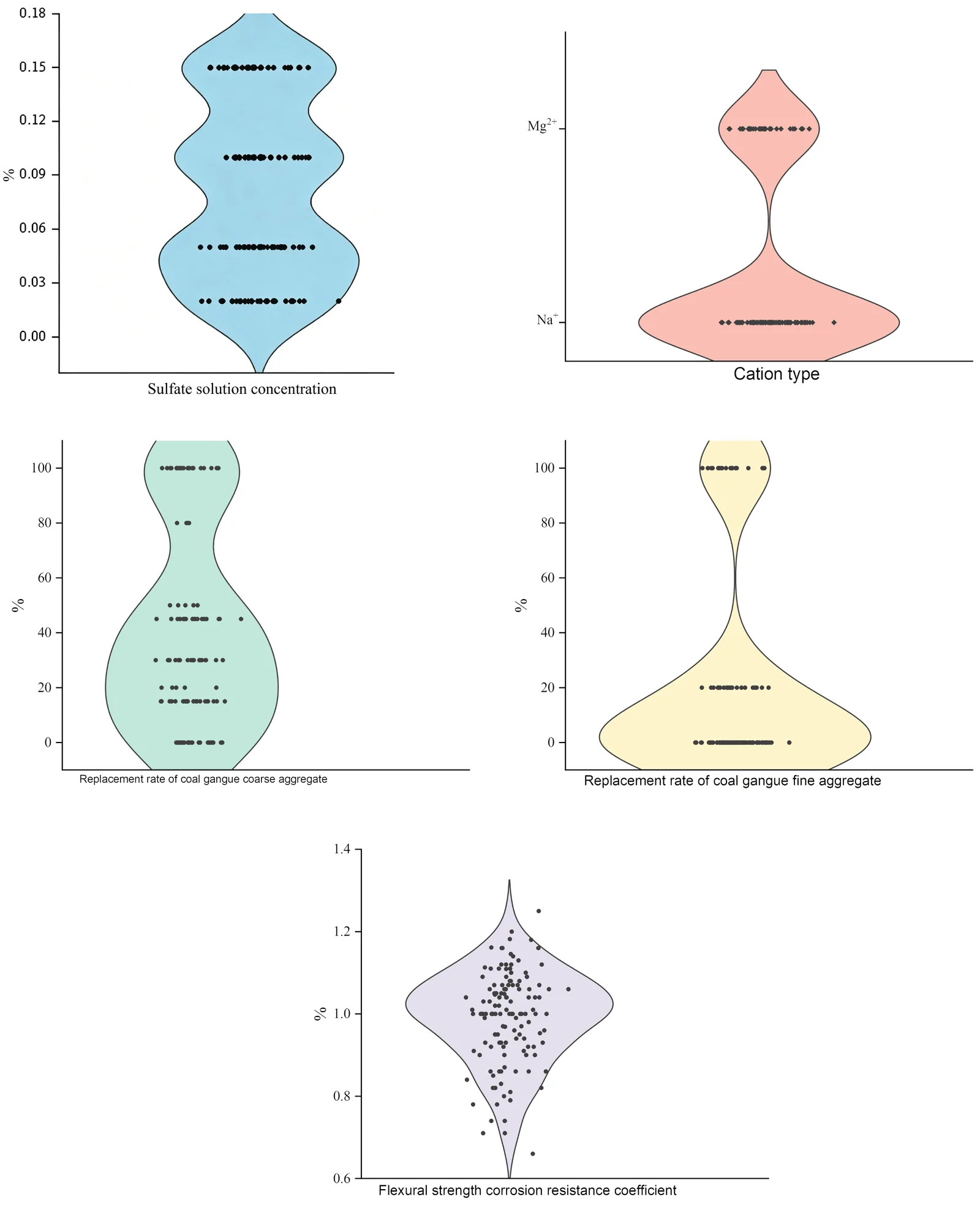
Data distribution diagram of flexural strength and corrosion resistance coefficient model.

Combining the experimental data obtained in this study with data extracted from three published references [13,35,40], a total of 133 datasets related to the flexural strength of CGC under sulfate attack were collected. Table 23 provides a statistical description of the input and output features for the flexural strength corrosion resistance coefficient model. The input features include the water-to-binder ratio, dry-wet cycle age, sulfate solution concentration, coal gangue coarse aggregate replacement rate, coal gangue fine aggregate replacement rate, and cation type, totaling six input features. The output feature is the flexural strength corrosion resistance coefficient. Figure 20 shows the data distribution of the flexural strength corrosion resistance coefficient prediction model, illustrating the data density distribution across one or more interval variables in the dataset.

**Figure 21:**
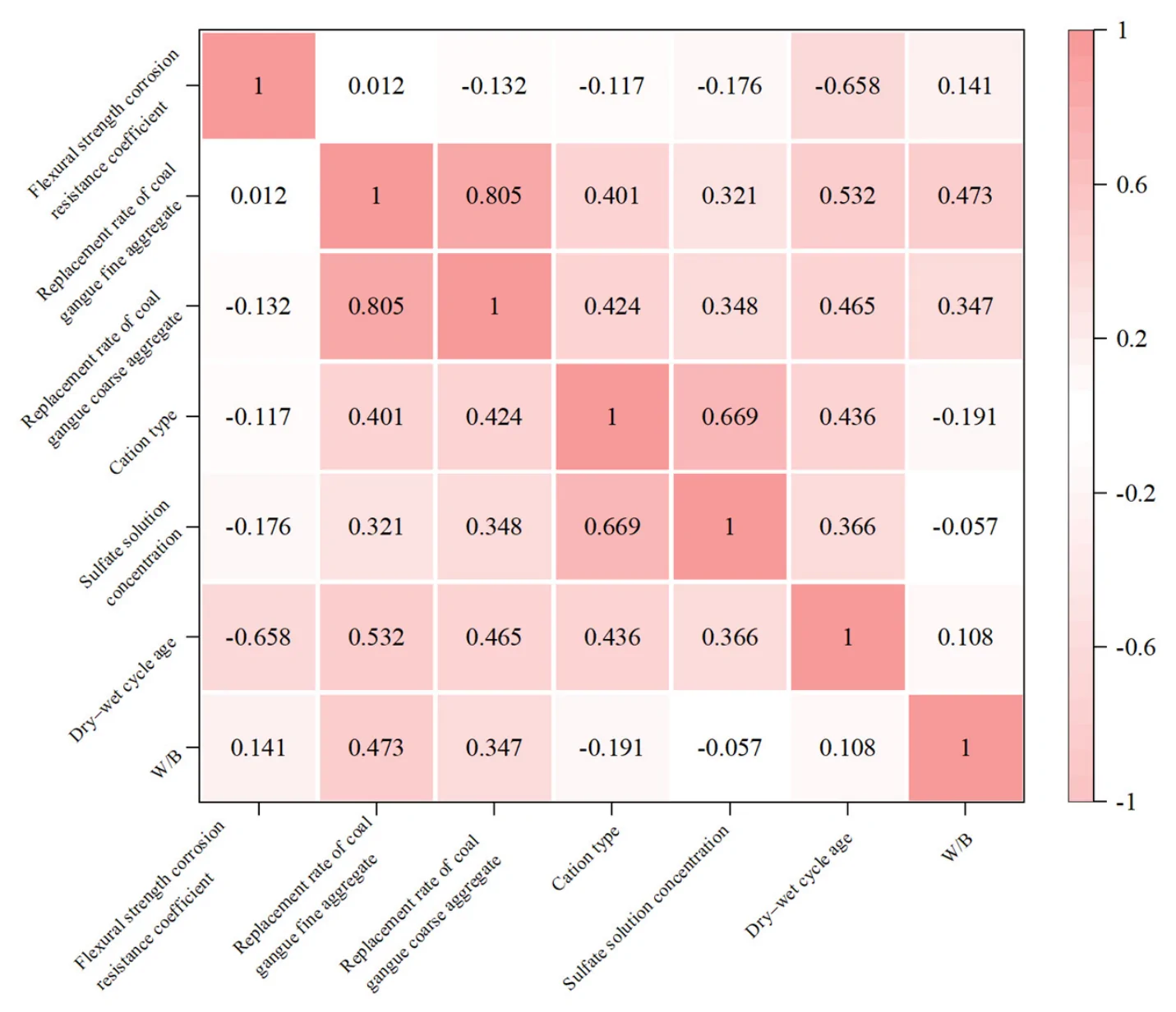
Pearson correlation diagram of flexural strength and corrosion resistance coefficient model.

The relationships between the input and output features were characterized using a Pearson correlation coefficient matrix, as shown in Figure 21. It can be seen from the figure that the dry-wet cycle age, sulfate solution concentration, and coal gangue coarse aggregate replacement rate are negatively correlated with the flexural strength corrosion resistance coefficient. Among them, the dry-wet cycle age exhibits the largest negative correlation coefficient, with a value of –0.658, indicating that compared with the other input features, the dry-wet cycle age has a considerable impact on the reduction of the flexural strength of concrete.

##### Determination of Hyperparameters for a Flexural Strength Corrosion Resistance Coefficient Mode

Hyperparameters of the machine learning models were optimized through an iterative approach, with each model undergoing multiple rounds of adjustment and iteration. The hyperparameter values for the flexural strength corrosion resistance coefficient model are presented in Table 24.

## Figures and Tables

**Table 1:** Morphological changes of specimens under sulfate attack.

Number of Dry-Wet Cycles (d)	Test Photos	Apparent Morphology Analysis
0	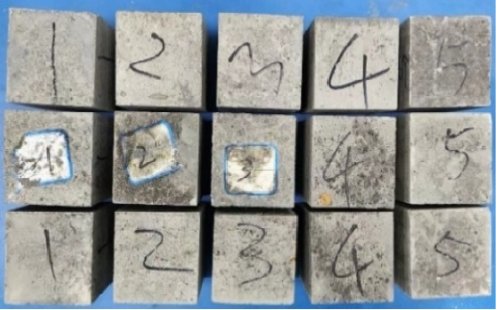	The specimens had flat and smooth surfaces with a few pores and no cracks. No spalling was observed at the edges and corners, and the overall shapes of the specimens remained intact.
30	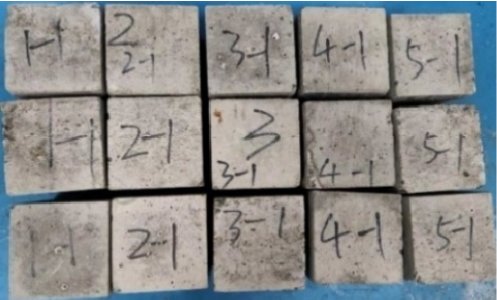	White powder was deposited around the pores on the specimen surfaces. Slight spalling occurred at the edges and corners, yet no internal aggregate was exposed, and the overall shapes of the specimens remained relatively intact.
60	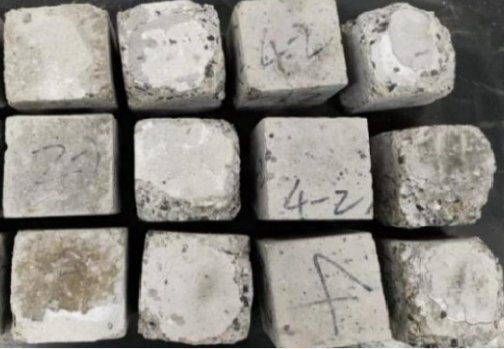	A salt crust developed on the specimen surfaces, and the specimens appeared white. The surface layer exhibited spalling, and pronounced corner spalling occurred around the edges and corners, exposing a small amount of internal aggregate. Fine cracks were also present on the surfaces.
90	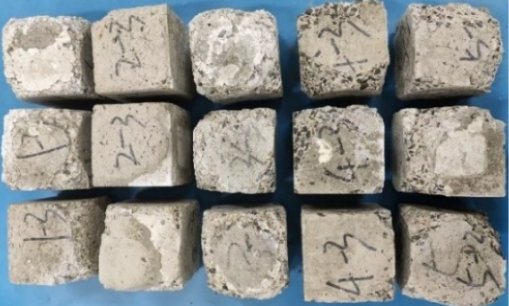	The edges and corners of the specimens were sanded, and severe aggregate spalling occurred around the specimens, exposing the internal aggregate. The salt crust on the specimen surfaces turned black, and the surface layer became loose and friable. Surface cracks propagated into the interior, and the originally straight edges and corners turned into curved surfaces.
120	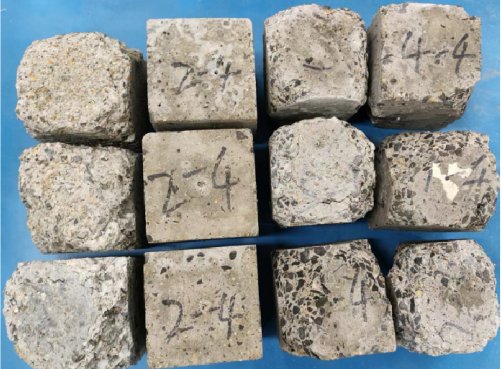	The specimens were severely corroded. Surface cracks penetrated through them, the entire body became loose and friable, the surface layer spalled off, massive aggregate spalling occurred at the corners with severe corner loss, the originally right-angled edges turned into curved surfaces, and the specimens lost their integrity.

**Table 2:** Mass of CGC specimens (Unit: g).

Number of Dry-Wet Cycles (d)	0	30	60	90	120
A-0-20	2341	2363	2367	2364	2337
A-20-20	2338	2365	2369	2361	2332
A-50-20	2337	2367	2370	2357	2324
A-80-20	2340	2371	2376	2353	2310

**Table 3:** Mass loss rate of CGC specimens.

Number of Dry-Wet Cycles (d)	0	30	60	90	120
A-0-20	0	0.941	1.114	0.981	−0.171
A-20-20	0	1.139	1.326	0.976	−0.256
A-50-20	0	1.282	1.403	0.852	−0.556
A-80-20	0	1.327	1.521	0.543	−1.269
A-100-20	0	1.543	1.629	0.134	−1.367

**Table 4:** Relative dynamic elastic modulus of CGC specimens.

Number of Dry-Wet Cycles (d)	0	30	60	90	120
A-0-20	1	1.012	1.027	0.991	0.811
A-20-20	1	1.027	1.039	0.973	0.901
A-50-20	1	1.041	1.046	0.981	0.863
A-80-20	1	1.037	1.062	0.953	0.971
A-100-20	1	1.061	1.086	0.891	0.781

**Table 5:** Compressive strength of CGC specimens under dry-wet cycling (Unit: MPa).

Number of Dry-Wet Cycles (d)	0	30	60	90	120
A-0-20	42.5	45.3	43.2	41.1	37.9
A-20-20	45.3	50.1	47.9	44.7	41.1
A-50-20	44.2	47.5	47.2	43.6	37.2
A-80-20	42.8	44.6	43.6	40.8	37.6
A-100-20	40.8	42.3	40.6	37.8	32.2

**Table 6:** Compressive strength corrosion resistance coefficient of CGC specimens.

Number of Dry-Wet Cycles (d)	0	30	60	90	120
A-0-20	1	1.066	1.016	0.966	0.891
A-20-20	1	1.105	1.057	0.987	0.907
A-50-20	1	1.075	1.067	0.986	0.842
A-80-20	1	1.043	1.018	0.954	0.879
A-100-20	1	1.037	0.995	0.927	0.788

**Table 7:** Flexural strength of CGC specimens under dry-wet cycles (Unit: MPa).

Number of Dry-Wet Cycles (d)	0	30	60	90	120
A-0-20	7.5	7.9	7.5	6.8	6.1
A-20-20	7.6	8.3	7.1	6.5	6.0
A-50-20	6.8	7.3	6.3	5.6	4.9
A-80-20	5.9	6.2	5.1	4.6	4.2
A-100-20	5.5	5.8	4.5	4.0	3.6

**Table 8:** Flexural strength corrosion resistance coefficient of CGC specimens.

Number of Dry-Wet Cycles (d)	0	30	60	90	120
A-0-20	1	1.057	0.998	0.912	0.816
A-20-20	1	1.098	0.941	0.859	0.784
A-50-20	1	1.078	0.928	0.822	0.727
A-80-20	1	1.051	0.871	0.782	0.716
A-100-20	1	1.047	0.825	0.736	0.662

**Table 9:** Semi-quantitative analysis of phase contents based on XRD characteristic peak intensities.

Phase	2θ/(°)	0 Dry-Wet Cycle	*R*_0_	60 Dry-Wet Cycles	*R*_60_	Variation Trend
		*I*_0_		*I*_60_		
SiO_2_	26.66	12,515	/	17,319	/	Internal reference
C-S-H gel	29.48	823	0.066	1072	0.062	Slightly decreased
Aft	9.00	887	0.071	801	0.046	Slight fluctuation
Aft	16.00	534	0.043	487	0.028	Slight fluctuation
Aft	23.00	664	0.053	746	0.043	Significantly increased
Gypsum	11.50	679	0.054	598	0.035	Decreased
Gypsum	20.8	4489	0.359	2717	0.157	Significantly increased
Ca(OH)_2_	18.00	946	0.076	1017	0.059	Generally stable
Ca(OH)_2_	34.00	814	0.065	762	0.044	Slightly consumed

**Table 10:** Evaluation index of compressive strength corrosion resistance coefficient models.

Model	Stage	R^2^	RMSE	MSE	MAE	MAPE
DT	Training	0.831	0.044	0.002	0.025	0.034
	Testing	0.806	0.052	0.003	0.037	0.037
SVM	Training	0.817	0.048	0.002	0.030	0.011
	Testing	0.862	0.043	0.002	0.033	0.048
ANN	Training	0.849	0.042	0.002	0.024	0.031
	Testing	0.863	0.040	0.002	0.022	0.030
RF	Training	0.864	0.040	0.002	0.029	0.005
	Testing	0.911	0.032	0.001	0.026	0.002
PSO-ANN	Training	0.873	0.038	0.001	0.028	0.027
	Testing	0.898	0.030	0.001	0.021	0.021
PSO-SVM	Training	0.897	0.034	0.001	0.021	0.018
	Testing	0.912	0.030	0.001	0.023	0.002

**Table 11:** Comparison between predicted and measured values based on the trained PSO-SVM model.

W/B	Number of Dry-Wet Cycles (d)	Sulfate Solution Concentration (%)	Replacement Rate of Coal Gangue Coarse Aggregate (%)	Replacement Rate of Coal Gangue Fine Aggregate (%)	Predicted K_c_	Measured K_c_	MAPE
0.4	30	10	0	20	0.9025	1.066	−0.1534
0.4	60	10	0	20	0.9026	1.016	−0.1116
0.4	90	10	0	20	0.9025	0.966	−0.0657
0.4	120	10	0	20	0.9024	0.891	0.0128
0.4	30	10	20	20	0.9027	1.105	−0.1831
0.4	60	10	20	20	0.9026	1.057	−0.1461
0.4	90	10	20	20	0.9025	0.987	−0.0856
0.4	120	10	20	20	0.9025	0.907	−0.0050
0.4	30	10	50	20	0.9026	1.075	−0.1604
0.4	60	10	50	20	0.9026	1.067	−0.1541
0.4	90	10	50	20	0.9025	0.986	−0.0847
0.4	120	10	50	20	0.9025	0.842	−0.0719
0.4	30	10	80	20	0.9025	1.043	−0.1347
0.4	60	10	80	20	0.9025	1.018	−0.1135
0.4	90	10	80	20	0.9024	0.954	−0.0541
0.4	120	10	80	20	0.9025	0.879	0.0267
0.4	30	10	100	20	0.9026	1.037	−0.0130
0.4	60	10	100	20	0.9025	0.995	−0.0930
0.4	90	10	100	20	0.9025	0.927	−0.0264
0.4	120	10	100	20	0.9024	0.788	0.1452

**Table 12:** Model prediction performance for flexural strength and corrosion resistance coefficient.

Model	Stage	R^2^	RMSE	MSE	MAE	MAPE
DT	Training	0.842	0.045	0.002	0.025	0.034
	Testing	0.884	0.036	0.001	0.026	0.027
SVM	Training	0.838	0.044	0.002	0.029	0.004
	Testing	0.886	0.041	0.002	0.032	0.029
ANN	Training	0.908	0.035	0.001	0.025	0.026
	Testing	0.867	0.036	0.001	0.026	0.027
RF	Training	0.864	0.043	0.002	0.032	0.003
	Testing	0.883	0.031	0.001	0.023	0.001
PSO-ANN	Training	0.911	0.032	0.001	0.022	0.023
	Testing	0.921	0.032	0.001	0.026	0.027
PSO-SVM	Training	0.924	0.015	0.0002	0.011	0.081
	Testing	0.981	0.032	0.001	0.026	0.005

**Table 13:** Comparison between predicted and measured values based on the trained PSO-SVM model.

W/B	Number of Dry-Wet Cycles (d)	Sulfate Solution Concentration (%)	Replacement Rate of Coal Gangue Coarse Aggregate (%)	Replacement Rate of Coal Gangue Fine Aggregate (%)	Predicted K_f_	Measured K_f_	MAPE
0.4	30	10	0	20	0.88098	1.057	−0.1665
0.4	60	10	0	20	0.88098	0.998	−0.1172
0.4	90	10	0	20	0.88097	0.912	−0.0340
0.4	120	10	0	20	0.88098	0.816	0.0796
0.4	30	10	20	20	0.88099	1.098	−0.1976
0.4	60	10	20	20	0.88097	0.941	−0.0638
0.4	90	10	20	20	0.88098	0.859	0.0256
0.4	120	10	20	20	0.88099	0.784	0.1237
0.4	30	10	50	20	0.88098	1.078	−0.1828
0.4	60	10	50	20	0.88097	0.928	−0.0507
0.4	90	10	50	20	0.88098	0.822	0.0718
0.4	120	10	50	20	0.88098	0.727	0.2118
0.4	30	10	80	20	0.88099	1.051	−0.1618
0.4	60	10	80	20	0.88097	0.871	0.0114
0.4	90	10	80	20	0.88098	0.782	0.1266
0.4	120	10	80	20	0.88098	0.716	0.2304
0.4	30	10	100	20	0.88099	1.047	−0.1586
0.4	60	10	100	20	0.88097	0.825	0.0678
0.4	90	10	100	20	0.88098	0.736	0.1969
0.4	120	10	100	20	0.88097	0.662	0.3308

**Table 14:** Mix ratio design of CGC sulfate attack test.

Code	Water (kg/m^3^)	Cement (kg/m^3^)	Fly Ash (kg/m^3^)	Gravel (kg/m^3^)	Sand (kg/m^3^)	Coal Gangue Coarse Aggregate (kg/m^3^)	Coal Gangue Fine Aggregate (kg/m^3^)	Superplasticizer (g)
A-0-20	180	360	90	1023	669.6	0	167.4	7.2
A-20-20	180	360	90	818.4	669.6	204.6	167.4	5.4
A-50-20	180	360	90	511.5	669.6	511.5	167.4	6.3
A-80-20	180	360	90	204.6	669.6	818.4	167.4	5.8
A-100-20	180	360	90	0	669.6	1023	167.4	8.1

Here, “A” represents a water-to-binder ratio of 0.4. The first number from left to right indicates the coal gangue coarse aggregate replacement rate, and the second number indicates the coal gangue fine aggregate replacement rate. For example, “A-100-20” signifies a water-to-binder ratio of 0.4, a coal gangue coarse aggregate replacement rate of 100%, and a coal gangue fine aggregate replacement rate of 20%.

**Table 15:** Physical properties and chemical compositions of the cement.

Parameter	Specific Surface Area (m^2^/kg)	SO_3_ (%)	MgO (%)	Cl^−^ (%)	Loss on Ignition (%)	Setting Time (min)		Compressive Strength (MPa)	Flexural Strength (MPa)
						Initial Setting Time	Final Setting Time	3 Days	3 Days
Test value	347	2.46	3.22	0.033	3.18	200	360	23.5	5.2

**Table 16:** Performance index of fly ash.

Parameter	Fineness (Residue on 5 μm Square Mesh Sieve) (%)	Loss on Ignition (%)	Moisture Content (%)	Density (g/cm^3^)	Bulk Censity (g/cm^3^)
Test value	16	2.8	0.85	2.54	1.13

**Table 17:** Chemical composition of fly ash.

Chemical Composition	SiO_2_	CaO	SO_3_	Al_2_O_3_	Alkali Content	Free CaO	Cl^−^	Iron Content
Mass fraction(%)	45.1	5.5	2.1	24.2	1.2	0.85	0.015	0.86

**Table 18:** Main physical performance indexes of pebbles.

Parameter	Content of Elongated and Flaky Particles (%)	Silt Content (%)	Apparent Density (kg/m^3^)	Bulk Density (kg/m^3^)	Crushing Value (%)
Test value	16	2.8	0.85	2.54	1.13

**Table 19:** Physical properties of coal gangue coarse and fine aggregates.

Material	Bulk Density (kg/m^3^)	Particle Density (kg/m^3^)	Water Absorption (%)	Porosity (%)	Moisture Content (%)	Crushing Value (%)
Coal gangue coarse aggregate	1502	2742	6.9	46	2.0	21.8
Coal gangue fine aggregate	1465	2618	2.7	45	0.9	10.4

**Table 20:** Design of sulfate attack specimens.

Solution	Solution Concentration	Immersion Method	Specimen Size	Number of Dry-Wet Cycles (d)			
				30	60	90	120
MgSO_4_	10%	Full immersion	100 mm × 100 mm × 100 mm	15	15	15	3
			40 mm × 40 mm × 160 mm	15	15	15	15

**Table 21:** Statistical analysis of compressive strength and corrosion resistance coefficient model data.

Parameter	Unit	Minimum	Maximum	Mean	Median	Standard Deviation	CV (%)	Type
Water-to-binder ratio	-	0.35	0.50	0.44	0.45	0.05	10.3	input
Dry-wet cycle age	d	0	120	41.74	30.00	33.05	79.18	input
Sulfate solution concentration	%	2	15	8.22	10	4.09	49.71	input
Replacement rate of coal gangue coarse aggregate	%	0	100	34.87	20	33.54	96.18	input
Replacement rate of coal gangue fine aggregate	%	0	100	33.46	0	31.99	225.57	input
Compressive strength corrosion resistance coefficient	-	0.45	1.29	0.99	1.00	0.11	11.12	output

**Table 22:** Hyperparameters of the compressive strength corrosion resistance coefficient model.

Model	Parameter	Value
ANN	Activation function	sigmoid
	Transfer function	purelin
	Number of neurons	12
	Error function	mse
	Learning rate	0.01
	Maximum training epochs	1000
SVM	Kernel function (mapping)	rbf
	Distance error(epsilon)	0.01
	Kernel function parameter(gamma)	1.3
	Cost parameter(c)	1.3
DT	Minimum number of samples at a leaf node	2
	Feature selection method	mse
RF	Number of trees	80
	Minimum number of samples for an internal split	2
	Maximum number of leaf nodes	2
	Feature selection method	mse
	Number of features in a subset	auto
PSO-ANN	Population size	80
	Learning factor	[−1,1]
	Particle velocity	[−1,1]
	Particle position range	[−0.5,0.5]
	Maximum number of iterations	100
PSO-SVM	Population size	80
	Learning factor	[−1,1]
	Particle velocity	[−0.1,0.1]
	Particle position range	[−1,1]
	Maximum number of iterations	100

**Table 23:** Statistical analysis of flexural strength and corrosion resistance coefficient model data.

Parameter	Unit	Minimum	Maximum	Mean	Median	Standard Deviation	CV (%)	Type
Water-to-binder ratio	-	0.4	0.50	0.45	0.45	0.04	10.10	input
Dry-wet cycle age	d	0	120	39.88	30.00	35.57	89.42	input
Sulfate solution concentration	%	2	15	8.2	10	4.84	58.96	
Replacement rate of coal gangue coarse aggregate	%	0	100	41.65	30	35.95	86.31	input
Replacement rate of coal gangue fine aggregate	%	0	100	21.80	0	37.47	171.88	input
Flexural strength corrosion resistance coefficient	-	0.66	1.25	0.99	1.00	0.11	11.31	output

**Table 24:** Hyperparameters of flexural strength and corrosion resistance coefficient model.

Model	Parameter	Value
ANN	Activation function	sigmoid
	Transfer function	purelin
	Number of neurons	10
	Error function	mse
	Learning rate	0.01
SVM	Kernel function (mapping)	rbf
	Distance error (epsilon)	0.01
	Kernel function parameter (gamma)	1.4
	Cost parameter (c)	1.4
DT	Minimum number of samples at a leaf node	2
	Feature selection method	mse
RF	Number of trees	80
	Minimum number of samples for an internal split	2
	Maximum number of leaf nodes	2
	Feature selection method	mse
	Number of features in a subset	auto
PSO-ANN	Population size	60
	Learning factor	[−1,1]
	Particle velocity	[−1,1]
	Particle position range	[−1,1]
	Maximum number of iterations	100
PSO-SVM	Population size	60
	Learning factor	[−1,1]
	Particle velocity	[−0.5,0.5]
	Particle position range	[−1,1]
	Maximum number of iterations	100

## Data Availability

The data presented in this study are openly available in article.
